# Targets and Effects of Common Biocompounds of *Hibiscus sabdariffa* (Delphinidin-3-Sambubiosid, Quercetin, and Hibiscus Acid) in Different Pathways of Human Cells According to a Bioinformatic Assay

**DOI:** 10.3390/nu16040566

**Published:** 2024-02-19

**Authors:** Sergio R. Zúñiga-Hernández, Trinidad García-Iglesias, Monserrat Macías-Carballo, Alejandro Pérez-Larios, Yanet Karina Gutiérrez-Mercado, Gabriela Camargo-Hernández, Christian Martin Rodríguez-Razón

**Affiliations:** 1 Departamento de Ciencias de la Salud, Centro Universitario de los Altos, Universidad de Guadalajara, Tepatitlán de Morelos 47620, Mexico; gabriela.camargo@academicos.udg.mx; 2 Instituto de Investigación de Cáncer en la Infancia y Adolescencia, Departamento de Fisiología, Centro Universitario de Ciencias de la Salud, Guadalajara 44340, Mexico; trinidad.giglesias@academicos.udg.mx; 3 Instituto de Investigación en Ciencias Médicas, Centro Universitario de los Altos, Universidad de Guadalajara, Tepatitlán de Morelos 47620, Mexico; monserrat.macias@cualtos.udg.mx; 4 Laboratorio de Nanomateriales, Agua y Energia, Departamento de Ingenierias, Centro Universitario de los Altos, Tepatitlán de Morelos 47620, Mexico; alarios@cualtos.udg.mx; 5 Laboratorio Biotecnológico de Investigación y Diagnóstico, Departamento de Clínicas, Centro Universitario de los Altos, Tepatitlán de Morelos 47620, Mexico; yanet.gutierrez@academicos.udg.mx; 6 Laboratorio de Experimentación Animal (Bioterio), Departamento de Ciencias de la Salud, Centro Universitario de los Altos, Tepatitlán de Morelos 47620, Mexico

**Keywords:** delphinidin-3-sambubiosid, quercetin, hibiscus acid, bioinformatics, *Hibiscus sabdariffa*

## Abstract

The utilization of food as a therapeutic measure for various ailments has been a prevalent practice throughout history and across different cultures. This is exemplified in societies where substances like *Hibiscus sabdariffa* have been employed to manage health conditions like hypertension and elevated blood glucose levels. The inherent bioactive compounds found in this plant, namely, delphinidin-3-sambubioside (DS3), quercetin (QRC), and hibiscus acid (HA), have been linked to various health benefits. Despite receiving individual attention, the specific molecular targets for these compounds remain unclear. In this study, computational analysis was conducted using bioinformatics tools such as Swiss Target Prediction, ShinnyGo 0.77, KEGG, and Stringdb to identify the molecular targets, pathways, and hub genes. Supplementary results were obtained through a thorough literature search in PubMed. DS3 analysis revealed potential genetic alterations related to the metabolism of nitrogen and glucose, inflammation, angiogenesis, and cell proliferation, particularly impacting the PI3K-AKT signaling pathway. QRC analysis demonstrated interconnected targets spanning multiple pathways, with some overlap with DS3 analysis and a particular focus on pathways related to cancer. HA analysis revealed distinct targets, especially those associated with pathways related to the nervous system. These findings emphasize the necessity for focused research on the molecular effects of DS3, QRC, and HA, thereby providing valuable insights into potential therapeutic pathways.

## 1. Introduction

*Hibiscus sabdariffa*, a widely recognized botanical species in the regions of Asia and America, possesses immense popularity. Consequently, it is extensively employed in a diverse array of commodities, ranging from the infusion of distinct flavors into water to the formulation of facial creams [1]. Its popularity may be due to its potential health benefits, ranging from antihypertensive to anticarcinogenic effects, especially since this plant is rich in numerous biological compounds [2]. The plant’s matrix is abundant in a considerable number of biocompounds, which can fluctuate based on the prevailing atmospheric conditions in which *Hibiscus sabdariffa* is cultivated. In general, it comprises anthocyanins, organic acids, and other phenolic compounds [3]. These compounds exhibit diverse characteristics, ranging from vibrant colors such as blue, red, or purple [4] to their flavor and odor. In terms of their potential therapeutic impact, considerable evidence suggests that a majority, if not all, of these compounds, can influence various cells in mammals [5]. Several biological models have shown that the biocompounds of *Hibiscus sabdariffa* can alter the prognosis of various disease states, as some of these are directly related to changes in biological signaling pathways. For example, one anthocyanin that has sparked significant interest is delphinidin-3-sambubioside (DS3), which exhibits a notable presence within this specific plant [2]. DS3 has shown several of the beneficial effects briefly described before, especially as a hypotensive and hypolipidemic agent [6,7]. Another important compound from *Hibiscus sabdariffa* is quercetin (QRC), one of the most researched phenolic compounds that is not an anthocyanin and that has been promoted as a potential therapeutic agent, especially for its potential anti-cancer properties [8,9,10,11]. Finally, hibiscus acid (HA) is the most represented organic acid of this plant and plays a role in the flavor of *Hibiscus sabdariffa,* and while there is little information about the therapeutic effect of this acid in particular [12], other organic acids do show beneficial biological properties [13,14]. Nevertheless, even if there have been studies where the potential effects of DS3, QRC, and/or HA have been tested, there is still a limited quantity of evidence regarding the precise impact all these bioactive compounds have on the signaling pathways of human cells. Therefore, the primary aim of this study is to employ bioinformatic tools to elucidate the potential targets that delphinidin-3-sambubioside (D3S), quercetin (QRC), and hibiscus acid (HA) may have in human cells, link those targets with biological signaling pathways, and then describe the relation between each biological targets and check for any evidence that could verify the predictions given by the bioinformatic tools.

## 2. Materials and Methods

### 2.1. Bioinformatic Analysis

**Figure 1:**
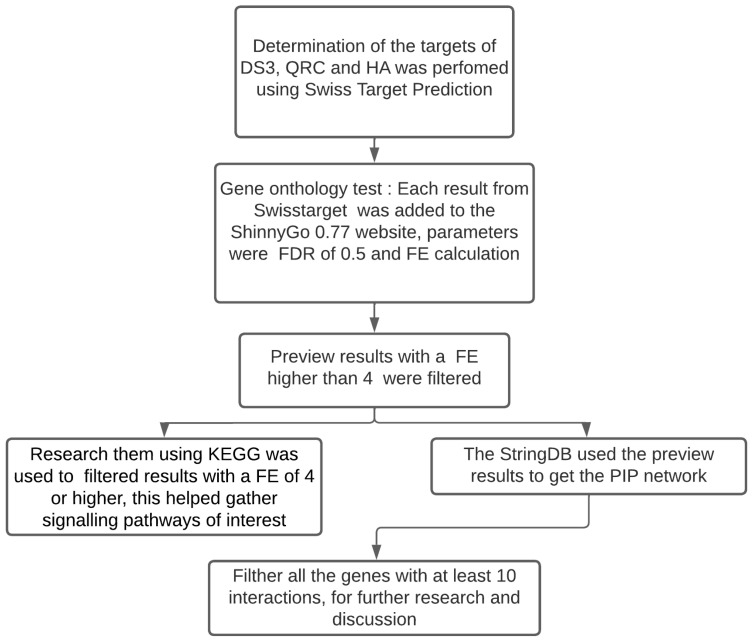
Bioinformatic analysis methodology diagram.

Delphinidin-3-sambubioside (D3S), quercetin (QRC), and hibiscus acid (HA) were chosen based on information from several articles that suggest those biocompounds are common among regional variants of *Hibiscus sabdariffa* [2,12]. The methodology for the following section of the study was a modification of an article published by Martinez-Esquivias et al. [15]. The SwissTargetPrediction website was utilized as a tool to identify potential molecular targets of DS3, QRC, and HA in human cells. Following this, a comprehensive list of potential targets was generated for each compound. The ShinnyGo 0.77 website was subsequently employed to compute the fold enrichment (FE) of each target, with a false discovery rate (FDR) threshold established at 0.05. Among these targets, those exhibiting an FE value greater than 4 were utilized to ascertain the pathways through which interactions induced by these biological compounds occur. This was accomplished through the utilization of the Kyoto Encyclopedia of Genes and Genomes (KEGG) database. Moreover, the Stringdb website was employed to retrieve the hub of genes derived from the FE data and to construct a Protein-to-Protein interaction network (PPI). This process is also shown in Figure 1. Finally, an extensive search was conducted using the Pubmed database to obtain relevant evidence.

### 2.2. Literature Search and Data Selection

A search was conducted on the PubMed database to ascertain pertinent articles that encompassed information about central genes acquired from bioinformatics analysis for D3S, QRC, and HA. These genes were generated through the utilization of uncomplicated search strings denoted as ‘gene/protein name’ and ‘biological compound name’. The search queries employed terms located within the title, abstract, or a fusion of both. Ultimately, inclusion and exclusion criteria were utilized to determine which articles could be incorporated into the final discussion.

### 2.3. Inclusion and Exclusion Criteria

The study’s inclusion criteria encompassed any research that examined genes or proteins derived from them, as identified in the bioinformatic analysis involving DS3, QRC, and/or HA. Conversely, the exclusion criteria were applied to studies with duplicated or overlapping data, papers exclusively presenting abstracts, conference proceedings, editorials, or author responses. Articles lacking full-text availability and systematic reviews were also excluded from consideration.

## 3. Results

### 3.1. Data from the Swiss Target Prediction

**Figure 2:**
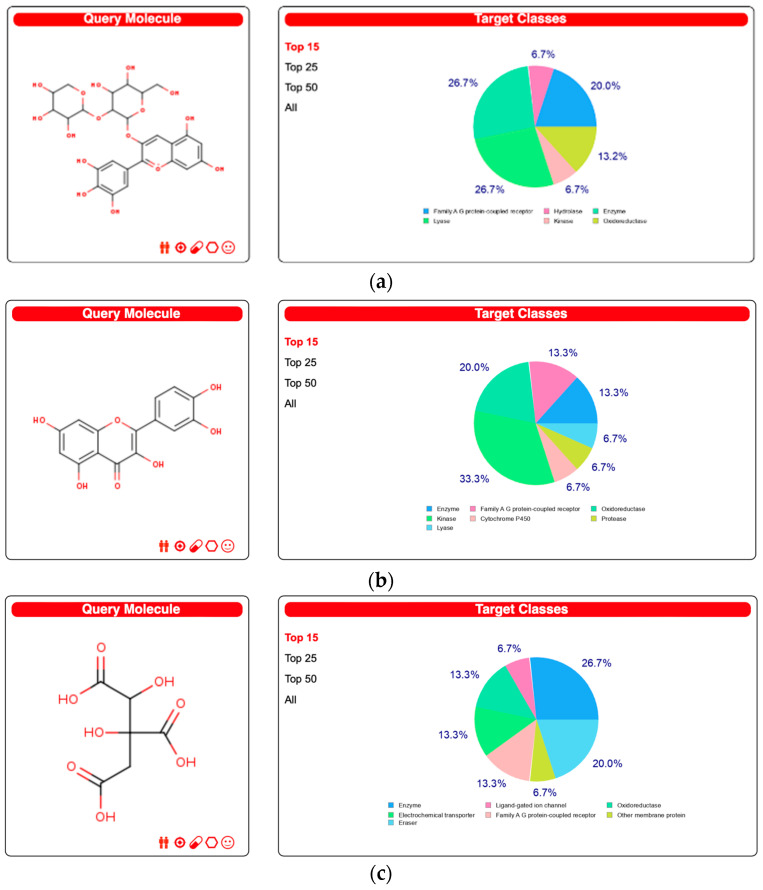
The top 15 molecular targets of the biocompounds according to the Swiss Target Prediction site. (**a**) DS3 Results, (**b**) QRC Results, and (**c**) HA Results.

Figure 2 presents a visual representation of the principal 15 target classes of molecules that can engage in interactions with each biological compound. Within DS3, these molecules are predominantly composed of enzymes and lyases, with a subsequent inclusion of a category encompassing G protein-coupled receptors. The bulk of targets associated with QRC are classified as oxidoreductases and kinases, whereas enzymes are the predominant targets within HA. For a comprehensive overview of all conceivable targets, Supplementary Material S1 offers complete information.

### 3.2. Analysis of Gene Ontology and Metabolic Pathways

The ShinnyGo 0.77 website was employed to execute the Gene Ontology and KEGG analyses. A comprehensive depiction of the gene ontology findings is presented in Table 1 for DS3, Table 2 for QRC, and Table 3 for HA. Furthermore, the KEGG outcomes are provided as visual representations of the respective pathway, highlighting the potential modifications in proteins as indicated in Supplementary Material S2.

### 3.3. Protein–Protein Interaction Network (PPI)

**Figure 3:**
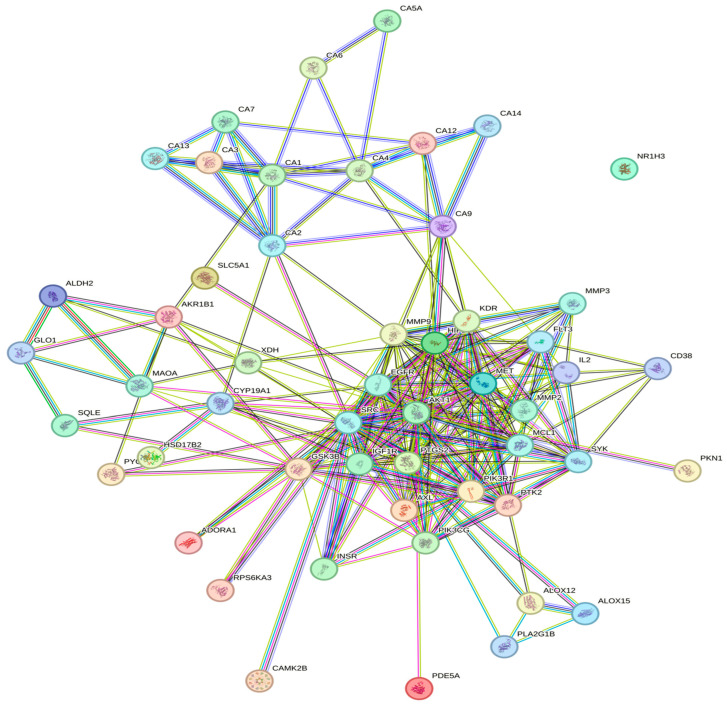
The Protein–Protein Interaction (PPI) network of DS3 is depicted, where each edge represents a specific protein with a notable protein–protein association. The blue and purple borders signify established interactions sourced from well-populated databases, including both previously curated and experimentally curated data. Predicted interactions within each neighborhood gene are differentiated by green, red, and navy-blue borders, corresponding to gene fusion, gene concurrence, and other predicted associations, respectively. Additional borders in grass green, black, and gray represent text-mining, co-expression, and protein homology, respectively.

**Figure 4:**
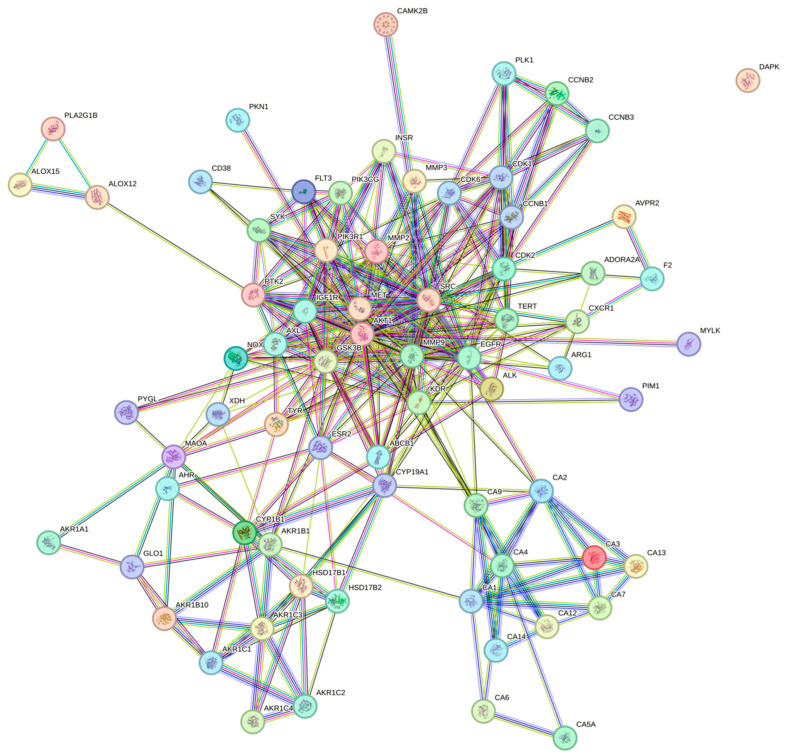
The PPI network of QRC is illustrated in the following description. Colored nodes signify both the query proteins and the first shell of interactors, while white nodes represent the second shell of interactors. Edges in the network represent protein–protein associations, and the colors denote the origin of these interactions. Specifically, a sky-blue color indicates information derived from curated databases, purple signifies experimentally determined interactions, and green, red, and navy blue indicate predicted interactions. Additionally, lime green, black, and light blue are used to represent associations sourced from text mining, co-expression, and protein homology, respectively.

**Figure 5:**
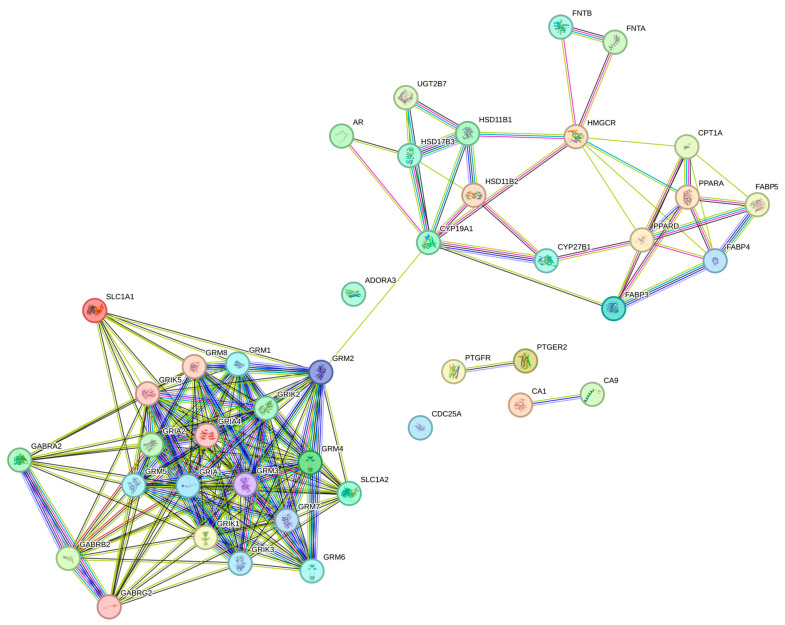
The PPI network of HA is outlined as follows: Colored nodes designate both the query proteins and the first shell of interactors, while white nodes represent the second shell of interactors. The edges in the network symbolize protein–protein associations, with colors indicating the origin of these interactions. A sky-blue color denotes information from curated databases, purple signifies experimentally determined interactions, and green, red, and navy blue indicate predicted interactions. Furthermore, lime green, black, and light blue are used to denote associations derived from text mining, co-expression, and protein homology, respectively.

The associations of protein targets for the three bioactive compounds were predicted using the STRING database, constructing a network with a medium confidence level of 0.400. Figure 3 illustrates the interactome for DS3, featuring 257 edges, 57 nodes, an average node degree of 9.02, and a highly significant PPI enrichment *p*-value of <1.0 × 10^−16^. Similarly, Figure 4 displays the interactome for QRC, comprising 328 edges, 71 nodes, an average node degree of 9.24, and a PPI enrichment *p*-value <1.0 × 10^−16^. Figure 5 exhibits the interactome for HA. Additionally, Table 4 lists the hub genes with at least 10 interactions for DS3, Table 5 outlines the hub genes meeting the same criterion for QRC, and Table 6 details the hub genes with at least 10 interactions for HA. 

### 3.4. Data Recollection from the Evidence of the Hub Genes with the Biocompounds

The results from the search in the PubMed database from the articles of the hub genes when tested in a study with DS3 and QRC are shown in Table 7 and Table 8, respectively. As for HA, no information about its relationship with any of the hub genes was found at the date of the data collection. These results will be notice if the evidence is found at the RNA, protein, or pathway level directly.

## 4. Discussion

The outcomes generated by the SwissTargetPrediction software (Figure 1) are rooted in the mathematical principles of the SwissTargetPrediction methodology [31]. This approach evaluates molecular binding targets by conducting a two-way analysis, comparing the molecular characteristics of the target molecules to others with similar attributes [32]; it conducts a physical 5D analysis of molecules, where three dimensions pertain to spatial considerations, and the remaining two dimensions involve atomic charge and lipophilicity, respectively. Additionally, SwissTargetPrediction incorporates a chemical comparison using the Tanimoto Index. Importantly, SwissTargetPrediction has proven valuable in the identification of molecular targets for small molecules derived from plants or foods, a context analogous to the focus of the current study. This underscores the versatility and applicability of SwissTargetPrediction in elucidating the molecular targets of bioactive compounds originating from natural sources. For example, in a study of 2022, a team of researchers from China led by Lili Yan [33] examined information on the predicted molecular targets of Erianin, a biphenyl compound, using this site. They proceeded to compare the identified targets, along with results from other bioinformatic tools, with the then-current information published by other authors. The comparison revealed a substantial overlap in their findings, illustrating the successful utilization of the SwissTargetPrediction tool to obtain molecular target information. 

In Table 1, the results from the Gene Ontology [34] assay show that DS3 affected several processes involving metabolism and inflammation; some of them are nitrogen metabolism, insulin resistance signaling, TNF signaling pathway, lipid and atherosclerosis, and endocrine resistance. As for Table 2, the Gene Ontology Assay shows that QRC affected other processes; while it also included metabolism and inflammation, it had plenty of differences, and those are Nitrogen metabolism, Ovarian steroidogenesis, Steroid hormone biosynthesis, EGFR tyrosine kinase inhibitor resistance, Endocrine resistance, Progesterone-mediated oocyte maturation, the ErbB signaling pathway, Prostate cancer, the FoxO signaling pathway, Chemical carcinogenesis, the Phospholipase D signaling pathway, Proteoglycans in cancer, Gastric cancer, Cellular senescence, Focal adhesion, MicroRNAs in cancer, the Rap1 signaling pathway, the PI3K-Akt signaling pathway, Pathways in cancer, and Metabolic pathways. Finally, the Gene Ontology assay of HA had interesting results; while some of them are similar to the ones in DS3 and QRC, it has different pathways not present in any of the previous compounds; it is intriguing that many of them are related to the nervous system somehow; all of the pathways are as follows: Nicotine addiction, Glutamatergic synapse, Terpenoid backbone biosynthesis, Nitrogen metabolism, Steroid hormone biosynthesis, the PPAR signaling pathway, Cocaine addiction, the Phospholipase D signaling pathway, the Neuroactive ligand-receptor interaction, Long-term potentiation, Retrograde endocannabinoid signaling, Long-term depression, Amphetamine addiction, Taste transduction, GABAergic synapse, Morphine addiction, Chemical carcinogenesis, the cAMP signaling pathway, Huntington disease, and Metabolic pathways. These findings are substantiated by the underlying principles of the database. The application of Fold Enrichment (FE) with a False Discovery Rate (FDR) cut-off of 0.05 is a well-established bioinformatic tool, widely accepted for delineating the likelihood of false positives [35]. These processes are related to a significant number of the effects described for DS3, QRC, or HA [2,3].

Concerning the KEGG [15,36] analysis on the impacted pathways, Table 1 (DS3) highlights a disturbance in the metabolism of nitrogen, a key element in energy metabolism and protein metabolism. Additionally, there is an indication of dysregulation of glucose metabolism (especially in muscle and adipose tissue). Interestingly, the glucose metabolism alterations seem to be attached mostly to alterations in the PI3K-Akt pathway; this could be explained by what Pal et al. found in a study looking for the effects of DS3 in non-small-cell Lung Cancer Cells (NSCLC) [18], by targeting EGRF/VEGFR2. In this study, EGFR, one of the targets predicted by bioinformatic tools, is inhibited when exposed to a dose of DS3 (going from 5–60 μM), generating a reduction in the expression of the PI3K subunits p85 and p110; this mechanism would reduce the phosphorylation of Akt, therefore reducing the effect of that signaling pathway. This result agrees with multiple studies that have studied the effects of DS3 in PI3K-AKT [16,17,18]. For QRC, Table 2 suggests various possible pathways and there is information that indicates such predictions could be the cause of some reported effects. Vaez et al. gave seventy-two women with polycystic ovary syndrome 500 mg of QRC; then they analyzed serum levels of LH hormone, FSH hormone, and IL-6 [37], and the authors concluded that QRC can decrease inflammatory and LH parameters. Results such as these also suggest that QRC has some implications for ovarian steroidogenesis just like the bioinformatic test predicts. Furthermore, QRC has been shown to affect nutrient metabolism; while the information provided by the KEGG analysis suggests changes in nitrogen metabolism and metabolic pathways, there is information published that may prove those predictions. For instance, Leyva-Soto et al. found in a randomized placebo-controlled study (*n* = 156) of patients with metabolic syndrome, when given a supplemented bread with QRC, that in comparison with controls, total cholesterol, LDL-cholesterol, total triglycerides, and fasting plasma glucose significantly decreased [38]; such result could be explained from changes in the metabolic pathways for these molecules. Nevertheless, there are some contradicting studies about this phenomenon. N. Arias et al. [39], in a study using Wistar rats, found no statistical significance in the reduction in the percentage of body fat for the rats with a high-fat, high-sucrose diet supplemented with QRC; they also showed no reduction in the expression of genes related to fatty acid oxidation like Peroxisome proliferator-activated receptor-alpha (PPAR-α), Peroxisome proliferator-activated-receptor-gamma-coactivator 1-alpha (PGC-1α), and CBT-1b.

Interestingly, those genes were not found to have an impact by the bioinformatic tools. Meanwhile, other studies have found that, in skeletal muscle, QRC can activate the adenosine monophosphate kinase (AMPK), thus increasing the translocation of the glucose-transporter type 4 (GLUT-4) receptor to the membrane, facilitating glucose uptake, and stimulating glycolysis [40]. These results may complement the information given by the bioinformatic tool for QRC up to this point. 

As for the results of KEGG for HA, while the results suggest it has an impact on pathways associated with the nervous system and others like PPAR, to date, there is little to no information regarding tests in the areas of most of the pathways described for HA. However, there has been some research regarding the impact of HA on rats, and the results have shown some potential therapeutic effects [26,27].

The PIP analysis [41,42,43,44,45,46,47,48] further reinforces the notion that the PI3K-AKT pathway is a primary target of DS3, with AKT emerging as the most interconnected node in the entire analysis. Additionally, insights from the hub genes (Table 2) indicate a consistent trend among genes, showcasing associations with glucose and nitrogen metabolism, inflammation, and angiogenesis. This alignment not only corroborates the earlier findings but also introduces angiogenesis, a process linked to nitric oxide production and, consequently, blood pressure. This is particularly noteworthy as one of the reported effects of DS3 is its potential as a hypotensive agent [6]. Nevertheless, information is absent regarding other potential targets that have been reported. For example, there is a precedent that DS3 can impact the cell cycle, by reducing the expression of Cycline D1 (key in the progression from the G1 to S phase in the cell cycle). Also, there is information that DS3 can also have an impact on apoptosis, by reducing anti-apoptotic protein transcription like BCL2, BCL-xL, and MCL-1 as well as increasing the activation of apoptotic proteins like Bak, Bax, Caspase 9, and Caspase 3 [16,18,49]. This could be explained by the low probability that plenty of the DS3 targets had in the SwissTargetPrediction software making, so those targets did not make the statistical cut for further in this analysis.

The PIP analysis for QRC is vastly different. In general, as Figure 4 shows, there are many more interactions between proteins than in the other two figures; even so, it seems that most of the interactions congregate around proteins related to cancer or inflammation in some way. Such results are interesting since plenty of the research available presents an interest in using QRC for cancer treatment. This is not so surprising since QRC has proved to have an anti-cancer effect [50], but the effects and targets can vary drastically depending on the tissue. For example, in 2019, Cing-Yu Chen et al. showed for the first time that QRC can blunt agonist-triggered Ca^2+^ signals by depleting the intracellular Ca^2+^ storage in mouse brain BEND.3 EC [51]. According to the authors, their results suggest that QRC can suppress vasodilation in the tissue and, therefore, explain how it is that it can help in combating glioma. Others, for example, have found the QRC effect to be explained (when used for cancer treatment) by its capacity to keep cancer cells in the G2/M phase and promote apoptosis. In 2022, Azizi et al. [52] used the breast cancer cell T47D with the CD44+/CD24− phenotype and assessed their cytotoxicity when exposed to QRC. Then, they analyzed the cell cycle distribution of the cells when exposed to QRC. Their results showed that, in a time-dependent matter, the half maximal inhibitory concentration (IC50) for QRC was almost 50 μM in 48 h for T47D cells, showing the anti-cancer effect of QRC; furthermore, when they analyzed the cell cycle distribution, they found an induced G2/M phase arrest in the T47D cells. The authors argue that this effect could be explained by increased expression of cyclin B and a reduced expression of cyclin D, E2F1, cyclin E, and E2F2 and thus, conclude that QRC reduced the number of viable cancer cells by this mechanism that is associated with apoptosis. Then, another group of authors found similar results but with a more resounding list of targets and mechanisms by which QRC has those effects. Srivastava et al. [53] found in an extensive study using several cancer cell lines that QRC is capable of triggering apoptosis in this kind of cell and, by Western blotting Nalm6 cells treated with QRC at different concentrations (0, 10, 20 μM for 24 h), found a level increase in apoptotic markers like p53 and MCL1 and a decrease in anti-apoptotic proteins such as BCL2 and BCL-xL. Furthermore, their results indicated the pathway activated for this process; in the mitochondrial intrinsic pathway, they detected the release of CYTOCHROME, MAC/DIABLO, Caspase 3, Caspase 9, and PARP1. Results like those previously discussed are of interest for this work since the bioinformatic tools agree with its predictions with many of their findings. In Stringdb alone, there is an agreement with the previously shown authors, especially with plenty of the targets of QRC in human cell lines, including cyclins, from Cycline-B1, Cycline-B2, and Cycline-B3, passing to Cycline-dependent kinase 1, 2, and 6, respectively, but this result also gives an indication that other key targets could intervene in these mechanisms, but there is not (to the date of this article) plenty of information about them and their relation with QRC, proteins like Serine/threonine-protein Kinase PLK1, Focal Adhesion Kinase 1, or the Proto-oncogene tyrosine-protein kinase SRC.

Finally, the protein-to-protein interaction (PIP) for HA is drastically different from the other two, and there is a greater separation between interactions where the focus relies upon the interactions of different glutamate-related proteins/receptors, but there is little if any information published about this interaction or any other. 

Regarding the evidence research, a search was conducted on the PubMed database to gather information on the hub genes identified through the protein-to-protein analysis and their relationship with each of the biocompounds. Notably, in the case of DS3, the findings are intriguing, particularly concerning its effects on EGFR. There is a wealth of evidence demonstrating that exposure to DS3 leads to a reduction in the expression of the EGFR gene, the suppression of the function of the protein transcribed from it, and ultimately, its association with the inhibition of the entire PI3K-AKT pathway under these conditions [13,16,18]. Nevertheless, it is crucial to highlight that, while many other genes exhibit predicted alterations, there is limited available information regarding their relationship with DS3, if any. Consequently, there is notable importance in conducting further studies to explore these genes in-depth. In the case of QRC, plenty of the interactions are correlated with pathways associated with cancer, and most of them influence the proteins, changing the phosphorylation effects of receptors like EFGR or part of pathways like PI3K [19,20,21,22,23,24,25,26]; even so, most of the hub genes gathered from the protein-to-protein analysis did not have any information available in Pub-Med; therefore, it is important to remark how much more information could be gathered in the future. Lastly, HA has no information in Pub-med regarding it and its hub genes/proteins. This is not surprising since, even if HA is the most abundant organic acid of *Hibiscus sabdariffa* being the acid with more percentage in this plant (going from 13 to 24%), it is the most lacking in research on comparison with others like citric and hidroxicitric acid [12].

## 5. Conclusions

The predictive analysis suggests that DS3 holds the potential to induce changes in genes associated with nitrogen and glucose metabolism, inflammation, angiogenesis, and cell proliferation. Notably, the available information indicates that these changes may occur not only at the mRNA level but also directly in the proteins. One of the most noteworthy outcomes from the bioinformatic tools is the potential modification in the function of numerous signaling pathways, particularly the impact of DS3 on the PI3K-AKT pathway through the inhibition of the EGFR receptor. These results align with and support the previously discussed findings.

However, it is crucial to note that there is a paucity of published studies on DS3 and its other potential targets, as suggested by the bioinformatic tools. The research in this area presents a significant opportunity for further investigation, holding a high probability of uncovering substantial insights into the mechanisms through which DS3 operates in human cells. In many instances, the same can be said about QRC; while this biocompound seems to have many more potential targets and has been more studied than any other, there is still much more to research about it, especially in its relationship with cancer treatment; while the currently available information shows that QRC has great apoptotic effects in cancer cells, the currently described mechanisms are still incomplete and also could vary drastically depending on the kind of cancer cell treated; this study gives a plethora of other targets that have not (to our knowledge) been tested and could be important for understanding its effects in human cells as a whole and not only cancer cells. Finally, the results of HA may be the most interesting ones; this compound is to our knowledge the least researched of all, and the results of the bioinformatic assay suggest that the effects of HA could be mostly on the nervous system, something that seems to be under-reported. In the end, it is important to remark that the results of this study suggest the need to generate more research to understand better the mechanisms and applications that not only DS3, QRC, or HA have in humans but also the natural products that contain them, such as *Hibiscus sabdariffa*.

This study predicts many targets for DS3, QRC, and HA, and those targets should be capable of modifying the nitrogen and glucose metabolism, inflammation, angiogenesis, proliferation, and cancer-related pathways. Furthermore, as the PIP showed, the relation between all the individual hub genes of each biocompound seems to be closely integrated. While currently there are published studies that suggest some of these predictions are accurate, to date, most potential therapeutic targets have not been researched, especially in the case of HA. As such, the information obtained through this study (just as any information obtained in in silico studies) opens a path for further research that takes into consideration the findings obtained from this paper, especially those testing experimentally the predictions here shown, not only for DS3, QRC, and HA but for whole natural products that contain them, such as *Hibiscus sabdariffa*.

## Figures and Tables

**Table 1:** Functional enrichment analysis of the genes predicting an interaction with DS3.

Enrichment FDR	nGenes	Pathway Genes	Fold Enrichment	Pathway	Genes
4.20 × 10^−18^	10	17	141.151703	Nitrogen metabolism	CA2 CA9 CA14 CA6 CA1 CA3 CA4 CA7 CA5A CA13
1.49 × 10^−9^	15	354	10.1677074	PI3K-Akt signaling pathway	GSK3B PIK3CG MET IL2 FLT3 PKN1 KDR IGF1R AKT1 MCL1 PIK3R1 EGFR SYK PTK2 INSR
2.21 × 10^−9^	9	79	27.3369754	EGFR tyrosine kinase inhibitor resistance	GSK3B MET KDR IGF1R AKT1 PIK3R1 EGFR AXL SRC
2.73 × 10^−9^	27	1527	4.24287044	Metabolic pathways	CD38 PTGS2 CA12 AKR1B1 HSD17B2 PYGL CA2 SQLE PIK3CG CA9 ALOX12 ALDH2 CA14 GLO1 CA6 CA1 CYP19A1 PDE5A XDH ALOX15 CA3 CA4 CA7 PLA2G1B CA5A CA13 MAOA
1.98 × 10^−7^	8	95	20.2069806	Endocrine resistance	MMP2 MMP9 IGF1R AKT1 PIK3R1 EGFR PTK2 SRC
3.94 × 10^−6^	7	108	15.5528265	Insulin resistance	NR1H3 GSK3B PYGL AKT1 PIK3R1 INSR RPS6KA3
4.89 × 10^−6^	6	70	20.5678196	Central carbon metabolism in cancer	HIF1 A MET FLT3 AKT1 PIK3R1 EGFR
2.28 × 10^−5^	8	214	8.97038859	Lipid and atherosclerosis	CAMK2B GSK3B MMP9 AKT1 PIK3R1 MMP3 PTK2 SRC
2.52 × 10^−5^	5	56	21.424812	Regulation of lipolysis in adipocytes	PTGS2 AKT1 PIK3R1 ADORA1 INSR
0.0001554	8	294	6.52946652	MAPK signaling pathway	MET FLT 3 KDR IGF1R AKT1 EGFR INSR RPS6KA3
0.00042661	5	112	10.712406	TNF signaling pathway	PTGS 2 MMP 9 AKT1 PIK3R1 MMP3
0.0008788	5	137	8.7575874	Insulin signaling pathway	GSK3 B PYGL AKT1 PIK3R1 INSR
0.00134156	5	155	7.74057725	Non-alcoholic fatty liver disease	NR1H 3 GSK 3B AKT1 PIK3R1 INSR
0.0024404	3	47	15.3164614	Carbohydrate digestion and absorption	SLC5A1 AKT1 PIK3R1
0.00373995	4	120	7.99859649	AMPK signaling pathway	IGF1R AKT1 PIK3R1 INSR
0.01950816	3	107	6.72779144	Glucagon signaling pathway	CAMK2B PYGL AKT1
0.02834824	2	46	10.432952	Type II diabetes mellitus	PIK3R1 INSR
0.02929805	2	47	10.2109742	Pyruvate metabolism	ALDH2 GLO1

**Table 2:** Functional enrichment analysis of the genes predicting an interaction with QRC.

Enrichment FDR	nGenes	Pathway Genes	Fold Enrichment	Pathway	Genes
4.83 × 10^−18^	10	17	138.241358	Nitrogen metabolism	CA2 CA9 CA14 CA6 CA1 CA3 CA4 CA7 CA5A CA13
3.54 × 10^−8^	7	51	32.256317	Ovarian steroidogenesis	HSD17B2 HSD17B1 CYP19A1 CYP1B1 IGF1R INSR AKR1C3
5.91 × 10^−9^	8	61	30.8210242	Steroid hormone biosynthesis	HSD17B2 HSD17B1 CYP19A1 CYP1B1 AKR1C2 AKR1C1 AKR1C3 AKR1C4
1.77 × 10^−9^	9	79	26.7733264	EGFR tyrosine kinase inhibitor resistance	GSK3B MET KDR IGF1R AKT1 PIK3R1 EGFR AXL SRC
6.38 × 10^−9^	9	95	22.2641346	Endocrine resistance	MMP2 MMP9 ESR2 IGF1R AKT1 PIK3R1 EGFR PTK2 SRC
9.14 × 10^−9^	9	100	21.1509278	Progesterone-mediated oocyte maturation	CDK2 CCNB1 IGF1R AKT1 PIK3R1 CCNB3 CCNB2 PLK1 CDK1
9.27 × 10^−7^	7	84	19.5841924	ErbB signaling pathway	CAMK2B GSK3B AKT1 PIK3R1 EGFR PTK2 SRC
1.38 × 10^−7^	8	97	19.3822935	prostate cancer	GSK3B MMP9 CDK2 IGF1R AKT1 PIK3R1 EGFR MMP3
6.03 × 10^−9^	10	131	17.9397183	FoxO signaling pathway	CDK2 CCNB1 IGF1R AKT1 PIK3R1 EGFR CCNB3 CCNB2 PLK1 INSR
4.57 × 10^−11^	14	223	14.7540104	Chemical carcinogenesis	NOX4 MET AHR AKR1A1 CYP1B1 AKT1 PIK3R1 EGFR AKR1C2 PTK2 AKR1C1 AKR1C3 SRC AKR1C4
1.98 × 10^−7^	9	148	14.2911675	Phospholipase D signaling pathway	PIK3CG AVPR2 AKT1 PIK3R1 EGFR CXCR1 SYK INSR F2
1.98 × 10^−8^	11	202	12.7975911	Proteoglycans in cancer	CAMK2B MMP2 MMP9 MET KDR IGF1R AKT1 PIK3R1 EGFR PTK2 SRC
2.87 × 10^−6^	8	148	12.70326	Gastric cancer	GSK3B ABCB1 MET CDK2 AKT1 PIK3R1 EGFR TERT
3.85 × 10^−6^	8	156	12.0518107	Cellular senescence	CDK6 CDK2 CCNB1 AKT1 PIK3R1 CCNB3 CCNB2 CDK1
1.98 × 10^−7^	10	200	11.7505155	Focal adhesion	MYLK GSK3B MET KDR IGF1R AKT1 PIK3R1 EGFR PTK2 SRC
4.65 × 10^−6^	8	161	11.6775309	MicroRNAs in cancer	ABCB1 MMP9 CDK6 MET PIM1 CYP1B1 PIK3R1 EGFR
3.35 × 10^−6^	9	210	10.0718704	Rap1 signaling pathway	MET KDR ADORA2A IGF1R AKT1 PIK3R1 EGFR INSR SRC
1.13 × 10^−9^	15	354	9.95806395	PI3K-Akt signaling pathway	GSK3B CDK6 PIK3CG MET FLT3 PKN1 CDK2 KDR IGF1R AKT1 PIK3R1 EGFR SYK PTK2 INSR
4.57 × 10^−11^	19	530	8.42489788	Pathways in cancer	CAMK2B GSK3B MMP2 MMP9 CDK6 MET FLT3 CDK2 PIM1 ESR2 IGF1R AKT1 PIK3R1 EGFR TERT PTK2 ALK F2 DAPK1
8.98 × 10^−12^	31	1527	4.77100169	Metabolic pathways	CD38 CA12 TYR AKR1B1 HSD17B2 PYGL CA2 PIK3CG CA9 HSD17B1 ALOX12 AKR1A1 CA14 ARG1 GLO1 CA6 CA1 CYP19A1

**Table 3:** Functional enrichment analysis of the genes predicting an interaction with HA.

Enrichment FDR	nGenes	Pathway Genes	Fold Enrichment	Pathway	Genes
2.73 × 10^−8^	6	40	55.1516129	Nicotine addiction	GABRG2 GRIA2 GABRB2 GABRA2 GRIA4 GRIA1
1.31 × 10^−23^	17	114	54.82908885	Glutamatergic synapse	GRIK5 SLC1A1 SLC1A2 GRM6 GRIA2 GRM4 GRIA4 GRM1 GRIA1 GRIK3 GRM2 GRIK2 GRM5 GRIK1 GRM8 GRM7 GRM3
0.000306466	3	22	50.13782991	Terpenoid backbone biosynthesis	HMGCR FNTA FNTB
0.005845035	2	17	43.25616698	Nitrogen metabolism	CA9 CA1
9.26 × 10^−6^	5	61	30.13749339	Steroid hormone biosynthesis	HSD11B1 HSD17B3 CYP19A1 UGT2B7 HSD11B2
8.88 × 10^−7^	6	75	29.41419355	PPAR signaling pathway	CPT1A PPARD FABP3 FABP5 FABP4 PPARA
0.002418552	3	49	22.51086241	Cocaine addiction	GRIA2 GRM2 GRM3
7.85 × 10^−9^	9	148	22.35876199	Phospholipase D signaling pathway	GRM6 PTGFR GRM4 GRM1 GRM2 GRM5 GRM8 GRM7 GRM3
2.25 × 10^−21^	21	350	22.06064516	Neuroactive ligand-receptor interaction	GRIK5 GRM6 GABRG2 GRIA2 PTGFR GRM4 PTGER2 GABRB2 GABRA2 GRIA4 GRM1 GRIA1 GRIK3 GRM2 GRIK2 GRM5 GRIK1 GRM8 GRM7 GRM3 ADORA3
0.000324344	4	67	21.95089071	Long-term potentiation	GRIA2 GRM1 GRIA1 GRM5
1.27 × 10^−7^	8	148	19.8744551	Retrograde endocannabinoid signaling	GABRG2 GRIA2 GABRB2 GABRA2 GRIA4 GRM1 GRIA1 GRM5
0.004067259	3	60	18.38387097	Long-term depression	GRIA2 GRM1 GRIA1
0.005712018	3	69	15.98597475	Amphetamine addiction	GRIA2 GRIA4 GRIA1
0.008700522	3	85	12.97685009	Taste transduction	GRM4 GABRA2 GRM1
0.009404411	3	89	12.39362088	GABAergic synapse	GABRG2 GABRB2 GABRA2
0.009520584	3	91	12.12123361	Morphine addiction	GABRG2 GABRB2 GABRA2
0.001753072	5	197	9.331914197	Chemical carcinogenesis	VDR CDC25A AR UGT2B7 PPARA
0.002418552	5	219	8.394461629	CAMP signaling pathway	GRIA2 PTGER2 GRIA4 GRIA1 PPARA
0.008334	5	306	6.00780097	Huntington’s disease	SLC1A2 GRIA2 GRIA4 GRIA1 GRM5
0.000129008	fifteen	1527	3.611762469 *	Metabolic pathways	FOLH1 HAO1 CA9 HMGCR HSD11B1 HSD17B3 ACLY CA1 CYP19A1 PGD PTGES G6PD UGT2B7 HSD11B2 AKR1B10

* The only value with a Fold Enrichment lower than 4 in the results.

**Table 4:** Hub genes with at least 10 interactions in humans were obtained from the predictions of interactions with delphinidin-3-sambubiosid (DS3).

Gene Symbol	Protein Name	Protein-Function
AKT1	RAC-alpha serine/threonine-protein kinase	Regulating a multitude of physiological processes, such as metabolism, cell proliferation, survival, growth, and angiogenesis.
PTK2	Focal adhesion kinase 1	Related to the increase in glucose uptake and glycogen synthesis in insulin-sensitive tissues.
IL2	Interleukin-2	Required for T-cell proliferation and other cells of the immune system.
PIK3R1	Phosphoinositide-3-kinase regulatory subunit alpha/beta/delta	Necessary for the insulin-stimulated increase in glucose uptake and glycogen synthesis.
SYK	Spleen-associated tyrosine kinase	Regulates biological processes, including immunity, cell adhesion, vascular development, and others.
PTGS2	Prostaglandin G/H synthase 2	Plays a role in the production of inflammatory prostaglandins.
MMP9	Matrix metalloproteinase-9	Key in local proteolysis of the extracellular matrix and leukocyte migration.
HIF1A	Hypoxia-inducible factor 1-alpha	Master transcriptional regulation in response to hypoxia.
MMP2	Matrix metalloproteinase-2 (gelatinase a)	Involved in angiogenesis, tissue repair, tumor invasion, inflammation, and atherosclerotic plaque rupture.
KDR	Vascular endothelial growth factor receptor 2	Essential in the regulation of angiogenesis, promotes the proliferation, survival, and migration of endothelial cells.
MET	Hepatocyte growth factor receptor	Regulates processes like proliferation, scattering, morphogenesis, and survival.
HGF	Hepatocyte growth factor	Growth factor for a broad spectrum of tissues and cell types.
EGFR	Epidermal growth factor receptor	Converts extracellular cues into appropriate cellular responses
IGF1R	Insulin-like growth factor 1 receptor	Involved in cell growth and survival control.
CA9	Carbonic anhydrase 9	Involved in pH regulation.
BLNK	B-cell linker protein	Important for the activation of NF-kappa-B and NFAT.

**Table 5:** Hub genes with at least 10 interactions in humans were obtained from the predictions of interactions with quercetin (QRC).

Gene Symbol	Protein Name	Protein-Function
ABCB1	ATP-dependent translocase	Translocates drugs and phospholipids across the membrane.
AHR	Aryl hydrocarbon receptor	Ligand-activated transcriptional activator.
AKR1A1	Aldo-keto reductase family 1 member A	Displays enzymatic activity toward endogenous metabolites such as aromatic and aliphatic aldehydes, ketones, monosaccharides, and bile acids, with a preference for negatively charged substrates, such as glucuronate and succinic semialdehyde.
AKR1B1	Aldo-keto reductase family 1 member B1	Displays enzymatic activity toward endogenous metabolites such as aromatic and aliphatic aldehydes, ketones, monosaccharides, and bile acids, with a preference for negatively charged substrates, such as glucuronate and succinic semialdehyde.
AKR1B10	Aldo-keto reductase family 1 member B10	Catalyzes the NADPH-dependent reduction in a wide variety of carbonyl-containing compounds to their corresponding alcohols.
AKR1C1	Aldo-keto reductase family 1 member C1	Converts progesterone to its inactive form, 20-alpha-dihydroxyprogesterone (20-alpha-OHP). In the liver and intestine, may have a role in the transport of bile.
AKR1C2	Aldo-keto reductase family 1 member C2	Works in concert with the 5-alpha/5-beta-steroid reductases to convert steroid hormones into the 3-alpha/5-alpha and 3-alpha/5-beta-tetrahydrosteroids.
AKR1C3	Aldo-keto reductase family 1 member C3	Catalyzes the conversion of aldehydes and ketones to alcohols. Catalyzes the reduction in prostaglandin (PG) D2, PGH2, and phenanthrenequinone (PQ) and the oxidation of 9-alpha,11-beta-PGF2 to PGD2.
AKR1C4	Aldo-keto reductase family 1 member C4	Catalyzes the transformation of the potent androgen dihydrotestosterone (DHT) into the less active form, 5-alpha-androstan- 3-alpha,17-beta-diol (3-alpha-diol).
AKT1	RAC-alpha serine/threonine-protein kinase	Regulate many processes including metabolism, proliferation, cell survival, growth, and angiogenesis. This is mediated through serine and/or threonine phosphorylation of a range of downstream substrates.
ALK	ALK tyrosine kinase receptor	Important role in the genesis and differentiation of the nervous system. Transduces signals from ligands at the cell surface, through specific activation of the mitogen-activated protein kinase (MAPK) pathway.
ALOX12	Arachidonate 12-lipoxygenase 12S-type	Mainly converts arachidonic acid to (12S)-hydroperoxyeicosatetraenoic acid/(12S)-HPETE but can also metabolize linoleic acid. In contrast does not react towards methyl esters of linoleic and arachidonic acids (by similarity).
ALOX15	Arachidonate 15-lipoxygenase	Non-heme iron-containing dioxygenase that catalyzes the stereo-specific peroxidation of free and esterified polyunsaturated fatty acids generating a spectrum of bioactive lipid mediators.
AXL	Tyrosine-protein kinase UFO receptor	Receptor tyrosine kinase that transduces signals from the extracellular matrix into the cytoplasm by binding growth factor GAS6 and that thus regulates many physiological processes including cell survival, cell proliferation, migration, and differentiation.
CA1-5,7,9,12-14	Carbonic anhydrases	Reversible hydration of carbon dioxide.
CAMK2B	Calcium/calmodulin-dependent protein kinase type II subunit beta	Calcium/calmodulin-dependent protein kinase that functions automatically after Ca(2+)/calmodulin-binding, it also generates autophosphorylation transport in skeletal muscle.
CCNB1	G2/mitotic-specific cyclin-B1	Essential for the control of the cell cycle at the G2/M (mitosis) transition.
CCNB2	G2/mitotic-specific cyclin-B2	Essential for the control of the cell cycle at the G2/M (mitosis) transition.
CCNB3	G2/mitotic-specific cyclin-B3	Plays an essential role in the control of the cell cycle, notably via their destruction during cell division. Its tissue specificity suggests that it may be required during early meiotic prophase I.
CDK1	Cyclin-dependent kinase 1	Cyclin-dependent kinase 1; plays a key role in the control of the eukaryotic cell cycle by modulating the centrosome cycle as well as mitotic onset; promotes G2-M transition and regulates G1 progress and G1-S transition via association with multiple interphase cyclins. Required for cells to entry into S-phase and mitosis.
CDK2,6	Cyclin-dependent kinase 2 and 6	Cyclin-dependent kinase 2; serine/threonine-protein kinase involved in the control of the cell cycle; essential for meiosis, but dispensable for mitosis.
CXCR1	CXC chemokine receptor type 1;	Receptor to interleukin-8, which is a powerful neutrophil chemotactic factor.
CYP19A1	Aromatase	A cytochrome P450 monooxygenase that catalyzes the conversion of C19 androgens, androst-4-ene-3,17-dione (androstenedione) and testosterone to the C18 estrogens, estrone and estradiol, respectively.
CYP1B1	Cytochrome P450 1B1	A cytochrome P450 monooxygenase involved in the metabolism of various endogenous substrates, including fatty acids, steroid hormones, and vitamins.
DAPK1	Death-associated protein kinase 1	Calcium/calmodulin-dependent serine/threonine kinase involved in multiple cellular signaling pathways that trigger cell survival, apoptosis, and autophagy.
EGFR	Epidermal growth factor receptor	Receptor tyrosine kinase binding ligands of the EGF family and activating several signaling cascades to convert extracellular cues into appropriate cellular responses.
ESR2	Estrogen receptor beta	Nuclear hormone receptor. Binds estrogens with an affinity similar to that of ESR1 and activates the expression of reporter genes containing estrogen response elements (ERE) in an estrogen-dependent manner.
FLT3	Receptor-type tyrosine-protein kinase FLT3	Tyrosine-protein kinase that acts as a cell-surface receptor for the cytokine FLT3LG and regulates the differentiation, proliferation, and survival of hematopoietic progenitor cells and dendritic cells.
GLO1	Lactoylglutathione lyase	Involved in the regulation of TNF-induced transcriptional activity of NF-kappa-B.
GSK3B	Glycogen synthase kinase-3 beta	Constitutively active protein kinase that acts as a negative regulator in the hormonal control of glucose homeostasis,
HSD17B1	Estradiol 17-beta-dehydrogenase 1	Estradiol 17-beta-dehydrogenase 1; favors the reduction in estrogens and androgens.
HSD17B2	Estradiol 17-beta-dehydrogenase 2	Estradiol 17-beta-dehydrogenase 2; capable of catalyzing the interconversion of testosterone and androstenedione, as well as estradiol and estrone.
IGF1R	Insulin-like growth factor 1 receptor alpha chain	Receptor tyrosine kinase that mediates the actions of insulin-like growth factor 1 (IGF1). Binds IGF1 with high affinity and IGF2 and insulin (INS) with a lower affinity.
INSR	Insulin receptor subunit alpha	Receptor tyrosine kinase that mediates the pleiotropic actions of insulin. The binding of insulin leads to the phosphorylation of several intracellular substrates, including insulin receptor substrates (IRS1, 2, 3, 4), SHC, GAB1, CBL, and other signaling intermediates.
KDR	Vascular endothelial growth factor receptor 2	Tyrosine-protein kinase that acts as a cell-surface receptor for VEGFA, VEGFC, and VEGFD. Plays an essential role in the regulation of angiogenesis, vascular development, vascular permeability, and embryonic hematopoiesis.
MAOA	Amine oxidase [flavin-containing] A	Catalyzes the oxidative deamination of biogenic and xenobiotic amines and has important functions in the metabolism of neuroactive and vasoactive amines in the central nervous system and peripheral tissues.
MET	Hepatocyte growth factor receptor	Receptor tyrosine kinase that transduces signals from the extracellular matrix into the cytoplasm by binding to hepatocyte growth factor/HGF ligand.
MMP2	72 kDa type IV collagenase	Ubiquitous metalloproteinase that is involved in diverse functions such as the remodeling of the vascular tissue, angiogenesis, tissue repair, tumor invasion, inflammation, and atherosclerotic plaque rupture.
MMP3	Stromelysin-1	Can degrade fibronectin, laminin, gelatins of type I, III, IV, and V, collagens III, IV, X, and IX, and cartilage proteoglycans. Activates procollagenase.
MMP9	67 kDa matrix metalloproteinase-9	May play an essential role in local proteolysis of the extracellular matrix and in leukocyte migration. Could play a role in bone osteoclastic resorption.
NOX4	NADPH oxidase 4	Constitutive NADPH oxidase that generates superoxide intracellularly upon the formation of a complex with CYBA/p22phox. Regulates signaling cascades probably through phosphatase inhibition.
PIK3CG	Phosphatidylinositol 4,5-bisphosphate 3-kinase catalytic subunit gamma isoform;	Phosphoinositide-3-kinase (PI3K) that phosphorylates PtdIns(4,5)P2 (Phosphatidylinositol 4,5-bisphosphate) to generate phosphatidylinositol 3,4,5-trisphosphate (PIP3)
PIK3R1	Phosphatidylinositol 3-kinase regulatory subunit alpha	Necessary for the insulin-stimulated increase in glucose uptake and glycogen synthesis in insulin-sensitive tissues
PLA2G1B	Phospholipase A2	PA2 catalyzes the calcium-dependent hydrolysis of the 2-acyl groups in 3-sn-phosphoglycerides; this releases glycerophospholipids and arachidonic acid that serve as the precursors of signal molecules.
PLK1	Serine/threonine-protein kinase PLK1	Serine/threonine-protein kinase that performs several important functions throughout the M phase of the cell cycle, including the regulation of centrosome maturation and spindle assembly, the removal of cohesins from chromosome arms, the inactivation of anaphase-promoting complex/cyclosome (APC/C) inhibitors, and the regulation of mitotic exit and cytokinesis.
PTK2	Focal adhesion kinase 1	Non-receptor protein-tyrosine kinase that plays an essential role in regulating cell migration, adhesion, spreading, reorganization of the actin cytoskeleton, the formation and disassembly of focal adhesions and cell protrusions, cell cycle progression, cell proliferation, and apoptosis.
PYGL	Glycogen phosphorylase, liver form	Phosphorylase is an important allosteric enzyme in carbohydrate metabolism.
CRS	Proto-oncogene tyrosine-protein kinase Src	Non-receptor protein tyrosine kinase that is activated following the engagement of many different classes of cellular receptors, including immune response receptors, integrins and other adhesion receptors, receptor protein tyrosine kinases, G protein-coupled receptors, as well as cytokine receptors.
SYK	Tyrosine-protein kinase SYK	Non-receptor tyrosine kinase that mediates signal transduction downstream of a variety of transmembrane receptors, including classical immunoreceptors like the B-cell receptor (BCR). Regulates several biological processes, including innate and adaptive immunity, cell adhesion, osteoclast maturation, platelet activation, and vascular development.
TERT	Telomerase reverse transcriptase	Telomerase is a ribonucleoprotein enzyme essential for the replication of chromosome termini in most eukaryotes. Active in progenitor and cancer cells. Inactive, or very low activity, in normal somatic cells.
TYR	Tyrosinase	This is a copper-containing oxidase that has a role in the formation of pigments such as melanin and other polyphenolic compounds. Also, catalyzes the initial and rate-limiting step in the cascade of reactions leading to melanin production from tyrosine.

**Table 6:** Hub genes with at least 10 interactions in humans were obtained from the predictions of interactions with hibiscus acid (HA).

Gene Symbol	Protein	Protein-Function
CPT1A	Carnitine O-palmitoyltransferase 1, liver isoform	Catalyzes the transfer of the acyl group of long-chain fatty acid-CoA conjugates onto carnitine, an essential step for the mitochondrial uptake of long-chain fatty acids and their subsequent beta-oxidation in the mitochondrion.
CYP19A1	Aromatase	A cytochrome P450 monooxygenase that catalyzes the conversion of C19 androgens, androst-4-ene-3,17-dione (androstenedione) and testosterone to the C18 estrogens, estrone and estradiol, respectively.
CYP27B1	25-hydroxyvitamin D-1 alpha hydroxylase, mitochondrial	A cytochrome P450 monooxygenase involved in vitamin D metabolism and in calcium and phosphorus homeostasis.
FABP3	Fatty acid-binding protein, heart	FABPs are thought to play a role in the intracellular transport of long-chain fatty acids and their acyl-CoA esters.
FABP4	Fatty acid-binding protein, adipocyte	Lipid transport protein in adipocytes. Binds both long-chain fatty acids and retinoic acid. Delivers long-chain fatty acids and retinoic acid to their cognate receptors in the nucleus. Belongs to the calycin superfamily. Fatty-acid binding protein (FABP) family.
FABP5	Fatty acid-binding protein 5	Intracellular carrier for long-chain fatty acids and related active lipids, such as the endocannabinoid, that regulates the metabolism and actions of the ligands they bind. In addition to the cytosolic transport, it selectively delivers specific fatty acids from the cytosol to the nucleus, wherein they activate nuclear receptors.
GABRA2	Gamma-aminobutyric acid receptor subunit alpha-2	A ligand-gated chloride channel that is a component of the heteropentameric receptor for GABA, the major inhibitory neurotransmitter in the brain (By similarity). Plays an important role in the formation of functional inhibitory GABAergic synapses in addition to mediating synaptic inhibition as a GABA-gated ion channel (by similarity).
GABRB2	Gamma-aminobutyric acid receptor subunit beta-2	A ligand-gated chloride channel that is a component of the heteropentameric receptor for GABA, the major inhibitory neurotransmitter in the brain. Plays an important role in the formation of functional inhibitory GABAergic synapses in addition to mediating synaptic inhibition as a GABA-gated ion channel.
GABRG2	Gamma-aminobutyric acid receptor subunit gamma-2	A ligand-gated chloride channel that is a component of the heteropentameric receptor for GABA, the major inhibitory neurotransmitter in the brain. Plays an important role in the formation of functional inhibitory GABAergic synapses in addition to mediating synaptic inhibition as a GABA-gated ion channel.
GRIA1	Glutamate receptor 1	Ionotropic glutamate receptor. L-glutamate acts as an excitatory neurotransmitter at many synapses in the central nervous system.
GRIA2	Glutamate receptor 2	Receptor for glutamate that functions as a ligand-gated ion channel in the central nervous system and plays an important role in excitatory synaptic transmission. L-glutamate acts as an excitatory neurotransmitter at many synapses in the central nervous system.
GRIA4	Glutamate receptor 4	Receptor for glutamate that functions as a ligand-gated ion channel in the central nervous system and plays an important role in excitatory synaptic transmission. L-glutamate acts as an excitatory neurotransmitter at many synapses in the central nervous system.
GRIK1	Glutamate ionotropic receptor, kainate 1	Ionotropic glutamate receptor. L-glutamate acts as an excitatory neurotransmitter in the central nervous system.
GRIK2	Glutamate ionotropic receptor, kainate 2	Ionotropic glutamate receptor. L-glutamate acts as an excitatory neurotransmitter at many synapses in the central nervous system.
GRIK3	Glutamate ionotropic receptor, kainate 3	Receptor for glutamate that functions as a ligand-gated ion channel in the central nervous system and plays an important role in excitatory synaptic transmission.
GRIK5	Ionotropic receptor glutamate, kainate 5	Receptor for glutamate. L-glutamate acts as an excitatory neurotransmitter at many synapses in the central nervous system.
GRM1-8	Metabotropic glutamate receptors 1 to 8	G-protein coupled receptor for glutamate.
HMGCR	3-hydroxy-3-methylglutaryl-coenzyme A reductase	Transmembrane glycoprotein that is the rate-limiting enzyme in cholesterol biosynthesis as well as in the biosynthesis of nonsterol isoprenoids that are essential for normal cell function including ubiquinone and geranylgeranyl proteins.
HSD11B1	Corticosteroid 11-beta-dehydrogenase isozyme 1	Catalyzes reversibly the conversion of cortisol to the inactive metabolite cortisone. Catalyzes reversibly the conversion of 7-ketocholesterol to 7-beta-hydroxycholesterol.
HSD11B2	Corticosteroid 11-beta-dehydrogenase isozyme 2	Catalyzes the conversion of cortisol to the inactive metabolite cortisone.
HSD17B3	Testosterone 17-beta-dehydrogenase 3	Favors the reduction in androstenedione to testosterone.
PPARA	Peroxisome proliferator-activated receptor alpha	Ligand-activated transcription factor. Key regulator of lipid metabolism. Activated by the endogenous ligand 1-palmitoyl-2-oleoyl-sn-glycerol-3-phosphocholine (16:0/18:1-GPC). Activated by oleylethanolamide, a naturally occurring lipid that regulates satiety.
PPARD	Peroxisome proliferator-activated receptor delta	Ligand-activated transcription factor. Receptor that binds peroxisome proliferators such as hypolipidemic drugs and fatty acids.
SLC1A1	Excitatory amino acid transporter 3	Sodium-dependent, high-affinity amino acid transporter that mediates the uptake of L-glutamate and also L-aspartate and D-aspartate.
SLC1A2	Excitatory amino acid transporter 2	Sodium-dependent, high-affinity amino acid transporter that mediates the uptake of L-glutamate and also L-aspartate and D-aspartate.
HSD17B3	Testosterone 17-beta-dehydrogenase 3	Favors the reduction in androstenedione to testosterone.
PPARA	Peroxisome proliferator-activated receptor alpha	Ligand-activated transcription factor. Key regulator of lipid metabolism.
PPARD	Peroxisome proliferator-activated receptor delta	Ligand-activated transcription factor. Receptor that binds peroxisome proliferators such as hypolipidemic drugs and fatty acids. Has a preference for poly-unsaturated fatty acids, such as gamma-linoleic acid and eicosapentaenoic acid.
SLC1A1	Excitatory amino acid transporter 3	Sodium-dependent, high-affinity amino acid transporter that mediates the uptake of L-glutamate and also L-aspartate and D-aspartate.
SLC1A2	Excitatory amino acid transporter 2	Sodium-dependent, high-affinity amino acid transporter that mediates the uptake of L-glutamate and also L-aspartate and D-aspartate.

**Table 7:** Evidence found of the Hub genes when tested against DS3.

Genes	Results at the Gene Expression Level	Results at the Protein Level	Results of Pathway Impact
MET		Deeba N. 2008 [16]: Suppress the phosphorylation of the protein.	
IGF1R		Teller et al., 2009 [17]: Inhibition of its kinase activity.	
EGFR	Harish Chandra Pal [18], et al., 2013: Reduction in the expression of the gene.	Fridrich D, et al., 2008 [13]: Suppress phosphorylation of the protein.	Harish Chandra Pal, et al., 2013 [18]: Inhibition of the PI3K-Akt pathway.

**Table 8:** The evidence found of the Hub genes when tested against QRC.

Genes	Results at the Gene Expression Level	Results at the Protein Level	Results of Pathway Impact
AHR		Mohammadi-Bardbori [19], 2012: Reduces protein activation.	Ciolino H, 1999 [20]: Generates changes in the pathway for Chemical carcinogenesis. Hamidullah 2015 [8]: Changes in the Akt-mTor pathway.
AK1RC3		Skarydová et al., 2014 [21]: Inhibition of the activity of the enzyme.	
CDK2		Chou, et al., 2010 [9]: Decreased levels of the protein	
CDK6	Mohd et al., 2020 [22]: Decreases the expression of the gene.	Teler et al., 2009 [13]: Inhibition of its kinase activity	
CYP1B1	Mense S, 2008 [23]: Increase in RNA transcription.	Mense S, 2008 [23]: Increase in levels of the protein in epithelial cells.	
EGFR		Yiqi et al., 2022 [24]: Decreases the phosphorylation of the protein/receptor. Huang Y, 1999 [25]: Decreases the phosphorylation of the protein/receptor.	
GSK3B		Chen K, et al., 2018 [26]: Decrease in the phosphorylation, promotes the activity of the protein.	
IGF1R		Wei-Jen, 2021 [27]: Reduces the phosphorylation of the protein.	
MAOA		Yauhen, 2014 [28]: The activity of the protein is decreased; it is probable that quercetin inhibits the protein; there is no decrease in the levels of protein.	
MET	Hui et al., 2015 [29]: Reduction on the transcription of the gene.	Hui et al., 2015 [29] Reduced phosphorylation of the protein.	
PIK3R1		Haitao et al., 2021 [30]: Inhibits the function of the protein	

## Data Availability

The original contributions presented in the study are included in the article and supplementary material, further inquiries can be directed to the corresponding authors.

## Supplementary Materials

The following supporting information can be downloaded at: https://www.mdpi.com/article/10.3390/nu16040566/s1, Supplementary Material S1: Most likely targets for DS3, QRC and HA; Supplementary Material S2: KEGG pathways for DS3, QRC and HA.
