# Supplementary material for: Targets and Effects of Common Biocompounds of Hibiscus sabdariffa (Delphinidin-3-Sambubiosid, Quercetin, and Hibiscus Acid) in Different Pathways of Human Cells According to a Bioinformatic Assay
Source: Nutrients. 2024 Feb 19;16(4):566. doi: 10.3390/nu16040566 (PMC10893457; doi:10.3390/nu16040566)
Supplement: Supplementary file 1 [file nutrients-16-00566-s001.zip › nutrients-2773844-supplementary.pdf]

Supplementary Material S1

Most likely targets for DS3

Table 11. Most likely targets for DS3

Target	Common name	Uniprot ID	ChEMBL ID	Target Class	Probability*	Known actives (3D/2D)
Lymphocyte differentiation antigen CD38	CD38	P28907	CHEMBL4660	Enzyme	0.109232505	0 / 1
Neuromedin-U receptor 2	NMUR2	Q9GZQ4	CHEMBL1075144	Family A G protein-coupled receptor	0.109232505	0 / 1
Alpha-2a adrenergic receptor	ADRA2A	P08913	CHEMBL1867	Family A G protein-coupled receptor	0.109232505	0 / 2
Adrenergic receptor alpha-2	ADRA2C	P18825	CHEMBL1916	Family A G protein-coupled receptor	0.109232505	0 / 10
Acetylcholinesterase	ACHE	P22303	CHEMBL220	Hydrolase	0.109232505	1 / 2
Aldose reductase (by homology)	AKR1B1	P15121	CHEMBL1900	Enzyme	0.100214944	0 / 48
NADPH oxidase 4	NOX4	Q9NPH5	CHEMBL1250375	Enzyme	0.100214944	0 / 4
Carbonic anhydrase II	CA2	P00918	CHEMBL205	Lyase	0.100214944	0 / 8
Carbonic anhydrase VII	CA7	P43166	CHEMBL2326	Lyase	0.100214944	0 / 10
Carbonic anhydrase XII	CA12	O43570	CHEMBL3242	Lyase	0.100214944	0 / 17
Carbonic anhydrase IV	CA4	P22748	CHEMBL3729	Lyase	0.100214944	0 / 8
Quinone reductase 2	NQO2	P16083	CHEMBL3959	Enzyme	0.09120885	0 / 19
Ribosomal protein S6 kinase alpha 3	RPS6KA3	P51812	CHEMBL2345	Kinase	0.09120885	0 / 1
Adenosine A1 receptor (by homology)	ADORA1	P30542	CHEMBL226	Family A G protein-coupled receptor	0.082221517	0 / 1
Cyclooxygenase-2	PTGS2	P35354	CHEMBL230	Oxidoreductase	0.082221517	0 / 12
Xanthine dehydrogenase	XDH	P47989	CHEMBL1929	Oxidoreductase	0.082221517	0 / 4
Phosphodiesterase 5A	PDE5A	O76074	CHEMBL1827	Phosphodiesterase	0.082221517	0 / 3
TNF-alpha	TNF	P01375	CHEMBL1825	Secreted protein	0.082221517	0 / 4
Interleukin-2	IL2	P60568	CHEMBL5880	Secreted protein	0.082221517	0 / 7
Cytochrome P450 1B1	CYP1B1	Q16678	CHEMBL4878	Cytochrome P450	0	0 / 11
Cytochrome P450 19A1	CYP19A1	P11511	CHEMBL1978	Cytochrome P450	0	0 / 7

Sodium/glucose cotransporter 2	SLC5A2	P31639	CHEMBL3884	Electrochemical transporter	0	0 / 10
Sodium/glucose cotransporter 1	SLC5A1	P13866	CHEMBL4979	Electrochemical transporter	0	0 / 3
Low affinity sodium-glucose cotransporter	SLC5A4	Q9NY91	CHEMBL1770047	Electrochemical transporter	0	0 / 2
Telomerase reverse transcriptase	TERT	O14746	CHEMBL2916	Enzyme	0	0 / 2
Squalene monooxygenase (by homology)	SQLE	Q14534	CHEMBL3592	Enzyme	0	1 / 0
Glyoxalase I	GLO1	Q04760	CHEMBL2424	Enzyme	0	1 / 0
Tyrosyl-DNA phosphodiesterase 1	TDP1	Q9NUW8	CHEMBL1075138	Enzyme	0	0 / 11
Myeloperoxidase	MPO	P05164	CHEMBL2439	Enzyme	0	0 / 1
PI3-kinase p85-alpha subunit	PIK3R1	P27986	CHEMBL2506	Enzyme	0	0 / 21
Liver glycogen phosphorylase	PYGL	P06737	CHEMBL2568	Enzyme	0	0 / 9
Estradiol 17-beta-dehydrogenase 2	HSD17B2	P37059	CHEMBL2789	Enzyme	0	0 / 1
Arachidonate 15-lipoxygenase	ALOX15	P16050	CHEMBL2903	Enzyme	0	0 / 1
PI3-kinase p110-gamma subunit	PIK3CG	P48736	CHEMBL3267	Enzyme	0	0 / 2
Arachidonate 12-lipoxygenase	ALOX12	P18054	CHEMBL3687	Enzyme	0	0 / 3
Phospholipase A2 group 1B	PLA2G1B	P04054	CHEMBL4426	Enzyme	0	0 / 1
DNA-(apurinic or apyrimidinic site) lyase	APEX1	P27695	CHEMBL5619	Enzyme	0	0 / 9
Aldo-keto reductase family 1 member C2 (by homology)	AKR1C2	P52895	CHEMBL5847	Enzyme	0	0 / 4
Lysine-specific demethylase 4D-like	KDM4E	B2RXH2	CHEMBL1293226	Eraser	0	0 / 9
Adenosine A3 receptor	ADORA3	P0DMS8	CHEMBL256	Family A G protein-coupled receptor	0	0 / 11
Dopamine D2 receptor (by homology)	DRD2	P14416	CHEMBL217	Family A G protein-coupled receptor	0	0 / 6
Adenosine A2a receptor (by homology)	ADORA2A	P29274	CHEMBL251	Family A G protein-coupled receptor	0	0 / 15

G-protein coupled receptor 35	GPR35	Q9HC97	CHEMBL1293267	Family A G protein-coupled receptor	0	0 / 1
Vasopressin V2 receptor	AVPR2	P30518	CHEMBL1790	Family A G protein-coupled receptor	0	0 / 2
Dopamine D4 receptor	DRD4	P21917	CHEMBL219	Family A G protein-coupled receptor	0	0 / 2
Interleukin-8 receptor A	CXCR1	P25024	CHEMBL4029	Family A G protein-coupled receptor	0	0 / 1
DNA topoisomerase II alpha	TOP2A	P11388	CHEMBL1806	Isomerase	0	0 / 1
Tyrosine-protein kinase SRC	SRC	P12931	CHEMBL267	Kinase	0	0 / 2
Epidermal growth factor receptor erbB1	EGFR	P00533	CHEMBL203	Kinase	0	0 / 1
Insulin-like growth factor I receptor	IGF1R	P08069	CHEMBL1957	Kinase	0	0 / 1
Tyrosine-protein kinase receptor FLT3	FLT3	P36888	CHEMBL1974	Kinase	0	0 / 1
Insulin receptor	INSR	P06213	CHEMBL1981	Kinase	0	0 / 1
Serine/threonine-protein kinase PIM1	PIM1	P11309	CHEMBL2147	Kinase	0	0 / 1
Serine/threonine-protein kinase Aurora-B	AURKB	Q96GD4	CHEMBL2185	Kinase	0	0 / 1
Myosin light chain kinase, smooth muscle	MYLK	Q15746	CHEMBL2428	Kinase	0	0 / 1
Death-associated protein kinase 1	DAPK1	P53355	CHEMBL2558	Kinase	0	0 / 1
Tyrosine-protein kinase SYK	SYK	P43405	CHEMBL2599	Kinase	0	0 / 2
Glycogen synthase kinase-3 beta	GSK3B	P49841	CHEMBL262	Kinase	0	0 / 2
Focal adhesion kinase 1	PTK2	Q05397	CHEMBL2695	Kinase	0	0 / 1
Vascular endothelial growth factor receptor 2	KDR	P35968	CHEMBL279	Kinase	0	0 / 2
Serine/threonine-protein kinase PLK1	PLK1	P53350	CHEMBL3024	Kinase	0	0 / 1
Cyclin-dependent kinase 1	CDK1	P06493	CHEMBL308	Kinase	0	0 / 1
Protein kinase N1	PKN1	Q16512	CHEMBL3384	Kinase	0	0 / 1
Casein kinase II alpha	CSNK2A1	P68400	CHEMBL3629	Kinase	0	0 / 1

Hepatocyte growth factor receptor	MET	P08581	CHEMBL3717	Kinase	0	0 / 1
Serine/threonine-protein kinase NEK2	NEK2	P51955	CHEMBL3835	Kinase	0	0 / 1
CaM kinase II beta	CAMK2B	Q13554	CHEMBL4121	Kinase	0	0 / 1
ALK tyrosine kinase receptor	ALK	Q9UM73	CHEMBL4247	Kinase	0	0 / 1
Serine/threonine-protein kinase AKT	AKT1	P31749	CHEMBL4282	Kinase	0	0 / 1
Serine/threonine-protein kinase NEK6	NEK6	Q9HC98	CHEMBL4309	Kinase	0	0 / 1
Tyrosine-protein kinase receptor UFO	AXL	P30530	CHEMBL4895	Kinase	0	0 / 1
NUAK family SNF1-like kinase 1	NUAK1	O60285	CHEMBL5784	Kinase	0	0 / 1
Carbonic anhydrase I	CA1	P00915	CHEMBL261	Lyase	0	0 / 1
Carbonic anhydrase IX	CA9	Q16790	CHEMBL3594	Lyase	0	0 / 1
Carbonic anhydrase XIII	CA13	Q8N1Q1	CHEMBL3912	Lyase	0	0 / 1
Carbonic anhydrase III	CA3	P07451	CHEMBL2885	Lyase	0	0 / 2
Carbonic anhydrase VI	CA6	P23280	CHEMBL3025	Lyase	0	0 / 1
Carbonic anhydrase XIV	CA14	Q9ULX7	CHEMBL3510	Lyase	0	0 / 1
Carbonic anhydrase VA	CA5A	P35218	CHEMBL4789	Lyase	0	0 / 1
Beta amyloid A4 protein	APP	P05067	CHEMBL2487	Membrane receptor	0	0 / 1
Sigma opioid receptor	SIGMAR1	Q99720	CHEMBL287	Membrane receptor	0	0 / 1
LXR-alpha	NR1H3	Q13133	CHEMBL2808	Nuclear receptor	0	0 / 1
Induced myeloid leukemia cell differentiation protein Mcl-1	MCL1	Q07820	CHEMBL4361	Other cytosolic protein	0	0 / 1
Arachidonate 5-lipoxygenase	ALOX5	P09917	CHEMBL215	Oxidoreductase	0	0 / 1
Aldehyde dehydrogenase	ALDH2	P05091	CHEMBL1935	Oxidoreductase	0	0 / 1
Monoamine oxidase A	MAOA	P21397	CHEMBL1951	Oxidoreductase	0	0 / 2
ATP-binding cassette sub-family G member 2	ABCG2	Q9UNQ0	CHEMBL5393	Primary active transporter	0	0 / 1
Multidrug resistance-associated protein 1	ABCC1	P33527	CHEMBL3004	Primary active transporter	0	0 / 1

P-glycoprotein 1 (by homology)	ABCB1	P08183	CHEMBL4302	Primary active transporter	0	0 / 1
Thrombin and coagulation factor X	F10	P00742	CHEMBL244	Protease	0	0 / 1
Plasminogen	PLG	P00747	CHEMBL1801	Protease	0	0 / 1
Thrombin	F2	P00734	CHEMBL204	Protease	0	0 / 1
Matrix metalloproteinase 13	MMP13	P45452	CHEMBL280	Protease	0	0 / 1
Matrix metalloproteinase 3	MMP3	P08254	CHEMBL283	Protease	0	0 / 1
Matrix metalloproteinase 9	MMP9	P14780	CHEMBL321	Protease	0	0 / 1
Matrix metalloproteinase 2	MMP2	P08253	CHEMBL333	Protease	0	0 / 2
Beta-secretase 1	BACE1	P56817	CHEMBL4822	Protease	0	0 / 1
Hypoxia-inducible factor 1 alpha	HIF1A	Q16665	CHEMBL4261	Transcription factor	0	0 / 1
Microtubule-associated protein tau	MAPT	P10636	CHEMBL1293224	Unclassified protein	0	0 / 1
Voltage-gated potassium channel subunit Kv1.3	KCNA3	P22001	CHEMBL4633	Voltage-gated ion channel	0	0 / 1

Most likely targets for QRC.

Table 12. Most likely targets of QRC

Target	Common name	Uniprot ID	ChEMBL ID	Target Class	Probability*	Known actives (3D/2D)
NADPH oxidase 4	NOX4	Q9NPH5	CHEMBL1250375	Enzyme	1.0	7 / 8
Vasopressin V2 receptor	AVPR2	P30518	CHEMBL1790	Family A G protein-coupled receptor	1.0	1 / 1
Aldose reductase	AKR1B1	P15121	CHEMBL1900	Enzyme	1.0	18 / 72

Xanthine dehydrogenase	XDH	P47989	CHEMBL1929	Oxidoreductase	1.0	12 / 20
Monoamine oxidase A	MAOA	P21397	CHEMBL1951	Oxidoreductase	1.0	4 / 14
Insulin-like growth factor I receptor	IGF1R	P08069	CHEMBL1957	Kinase	1.0	3 / 3
Tyrosine-protein kinase receptor FLT3	FLT3	P36888	CHEMBL1974	Kinase	1.0	5 / 7
Cytochrome P450 19A1	CYP19A1	P11511	CHEMBL1978	Cytochrome P450	1.0	5 / 18
Epidermal growth factor receptor erbB1	EGFR	P00533	CHEMBL203	Kinase	1.0	5 / 28
Thrombin	F2	P00734	CHEMBL204	Protease	1.0	11 / 3
Carbonic anhydrase II	CA2	P00918	CHEMBL205	Lyase	1.0	7 / 15
Serine/threonine-protein kinase PIM1	PIM1	P11309	CHEMBL2147	Kinase	1.0	7 / 7
Arachidonate 5-lipoxygenase	ALOX5	P09917	CHEMBL215	Oxidoreductase	1.0	5 / 46
Serine/threonine-protein kinase Aurora-B	AURKB	Q96GD4	CHEMBL2185	Kinase	1.0	3 / 4
Dopamine D4 receptor	DRD4	P21917	CHEMBL219	Family A G protein-coupled receptor	1.0	1 / 1
Adenosine A1 receptor (by homology)	ADORA1	P30542	CHEMBL226	Family A G protein-coupled receptor	1.0	6 / 23
Carbonic anhydrase VII	CA7	P43166	CHEMBL2326	Lyase	1.0	8 / 14
Glyoxalase I	GLO1	Q04760	CHEMBL2424	Enzyme	1.0	3 / 4
Myeloperoxidase	MPO	P05164	CHEMBL2439	Enzyme	1.0	1 / 1
PI3-kinase p85-alpha subunit	PIK3R1	P27986	CHEMBL2506	Enzyme	1.0	1 / 1
Adenosine A2a receptor (by homology)	ADORA2A	P29274	CHEMBL251	Family A G protein-coupled receptor	1.0	5 / 11
Death-associated protein kinase 1	DAPK1	P53355	CHEMBL2558	Kinase	1.0	2 / 2
Liver glycogen phosphorylase	PYGL	P06737	CHEMBL2568	Enzyme	1.0	1 / 1
Carbonic anhydrase I	CA1	P00915	CHEMBL261	Lyase	1.0	3 / 5
Glycogen synthase kinase-3 beta	GSK3B	P49841	CHEMBL262	Kinase	1.0	3 / 6
Tyrosine-protein kinase SRC	SRC	P12931	CHEMBL267	Kinase	1.0	2 / 10
Focal adhesion kinase 1	PTK2	Q05397	CHEMBL2695	Kinase	1.0	1 / 2
Estradiol 17-beta-dehydrogenase 2	HSD17B2	P37059	CHEMBL2789	Enzyme	1.0	8 / 3

Vascular endothelial growth factor receptor 2	KDR	P35968	CHEMBL279	Kinase	1.0	2 / 3
Matrix metalloproteinase 13	MMP13	P45452	CHEMBL280	Protease	1.0	1 / 1
Matrix metalloproteinase 3	MMP3	P08254	CHEMBL283	Protease	1.0	1 / 1
Carbonic anhydrase III	CA3	P07451	CHEMBL2885	Lyase	1.0	1 / 1
Arachidonate 15-lipoxygenase	ALOX15	P16050	CHEMBL2903	Enzyme	1.0	4 / 8
Multidrug resistance-associated protein 1	ABCC1	P33527	CHEMBL3004	Primary active transporter	1.0	7 / 11
Serine/threonine-protein kinase PLK1	PLK1	P53350	CHEMBL3024	Kinase	1.0	2 / 3
Carbonic anhydrase VI	CA6	P23280	CHEMBL3025	Lyase	1.0	1 / 1
Cyclin-dependent kinase 1	CDK1	P06493	CHEMBL308	Kinase	1.0	2 / 13
Matrix metalloproteinase 9	MMP9	P14780	CHEMBL321	Protease	1.0	2 / 2
Carbonic anhydrase XII	CA12	O43570	CHEMBL3242	Lyase	1.0	8 / 17
Matrix metalloproteinase 2	MMP2	P08253	CHEMBL333	Protease	1.0	2 / 2
Protein kinase N1	PKN1	Q16512	CHEMBL3384	Kinase	1.0	1 / 3
Carbonic anhydrase XIV	CA14	Q9ULX7	CHEMBL3510	Lyase	1.0	1 / 1
Carbonic anhydrase IX	CA9	Q16790	CHEMBL3594	Lyase	1.0	3 / 5
Casein kinase II alpha	CSNK2A1	P68400	CHEMBL3629	Kinase	1.0	3 / 2
Arachidonate 12-lipoxygenase	ALOX12	P18054	CHEMBL3687	Enzyme	1.0	5 / 10
Hepatocyte growth factor receptor	MET	P08581	CHEMBL3717	Kinase	1.0	2 / 4
Carbonic anhydrase IV	CA4	P22748	CHEMBL3729	Lyase	1.0	7 / 13
Serine/threonine-protein kinase NEK2	NEK2	P51955	CHEMBL3835	Kinase	1.0	1 / 2
Interleukin-8 receptor A	CXCR1	P25024	CHEMBL4029	Family A G protein-coupled receptor	1.0	1 / 1
CaM kinase II beta	CAMK2B	Q13554	CHEMBL4121	Kinase	1.0	1 / 2
ALK tyrosine kinase receptor	ALK	Q9UM73	CHEMBL4247	Kinase	1.0	2 / 4
Serine/threonine-protein kinase AKT	AKT1	P31749	CHEMBL4282	Kinase	1.0	1 / 4
P-glycoprotein 1	ABCB1	P08183	CHEMBL4302	Primary active transporter	1.0	11 / 48
Serine/threonine-protein kinase NEK6	NEK6	Q9HC98	CHEMBL4309	Kinase	1.0	1 / 2
Phospholipase A2 group 1B	PLA2G1B	P04054	CHEMBL4426	Enzyme	1.0	1 / 1

Carbonic anhydrase VA	CA5A	P35218	CHEMBL4789	Lyase	1.0	1 / 1
Beta-secretase 1	BACE1	P56817	CHEMBL4822	Protease	1.0	8 / 14
Cytochrome P450 1B1	CYP1B1	Q16678	CHEMBL4878	Cytochrome P450	1.0	12 / 46
Tyrosine-protein kinase receptor UFO	AXL	P30530	CHEMBL4895	Kinase	1.0	2 / 4
ATP-binding cassette sub-family G member 2	ABCG2	Q9UNQ0	CHEMBL5393	Primary active transporter	1.0	6 / 50
NUAK family SNF1-like kinase 1	NUAK1	O60285	CHEMBL5784	Kinase	1.0	1 / 2
Aldo-keto reductase family 1 member C2 (by homology)	AKR1C2	P52895	CHEMBL5847	Enzyme	1.0	1 / 1
Aldo-keto reductase family 1 member C1 (by homology)	AKR1C1	Q04828	CHEMBL5905	Enzyme	1.0	1 / 1
Aldo-keto-reductase family 1 member C3 (by homology)	AKR1C3	P42330	CHEMBL4681	Enzyme	1.0	1 / 1
Aldo-keto reductase family 1 member C4 (by homology)	AKR1C4	P17516	CHEMBL4999	Enzyme	1.0	1 / 1
Carbonic anhydrase XIII (by homology)	CA13	Q8N1Q1	CHEMBL3912	Lyase	1.0	1 / 1
Aldehyde reductase (by homology)	AKR1A1	P14550	CHEMBL2246	Enzyme	1.0	1 / 1
G-protein coupled receptor 35	GPR35	Q9HC97	CHEMBL1293267	Family A G protein-coupled receptor	1.0	2 / 4
Microtubule-associated protein tau	MAPT	P10636	CHEMBL1293224	Unclassified protein	0.68028359158	1 / 1
Lysine-specific demethylase 4D-like	KDM4E	B2RXH2	CHEMBL1293226	Eraser	0.68028359158	2 / 2
DNA topoisomerase II alpha	TOP2A	P11388	CHEMBL1806	Isomerase	0.68028359158	1 / 1
Insulin receptor	INSR	P06213	CHEMBL1981	Kinase	0.68028359158	1 / 1
Acetylcholinesterase	ACHE	P22303	CHEMBL220	Hydrolase	0.68028359158	4 / 27
Myosin light chain kinase, smooth muscle	MYLK	Q15746	CHEMBL2428	Kinase	0.68028359158	1 / 1
Tyrosine-protein kinase SYK	SYK	P43405	CHEMBL2599	Kinase	0.68028359158	3 / 3
PI3-kinase p110-gamma subunit	PIK3CG	P48736	CHEMBL3267	Enzyme	0.68028359158	3 / 1
DNA-(apurinic or apyrimidinic site) lyase	APEX1	P27695	CHEMBL5619	Enzyme	0.68028359158	1 / 1
Receptor-type tyrosine-protein phosphatase S	PTPRS	Q13332	CHEMBL2396508	Phosphatase	0.545871744949	6 / 8
Estrogen receptor beta	ESR2	Q92731	CHEMBL242	Nuclear receptor	0.545871744949	62 / 29

DNA-3-methyladenine glycosylase	MPG	P29372	CHEMBL3396943	Enzyme	0.545871744949	1 / 1
Solute carrier family 22 member 12	SLC22A12	Q96S37	CHEMBL6120	Electrochemical transporter	0.545871744949	4 / 1
Cyclin-dependent kinase 5/CDK5 activator 1	CDK5R1<br>CDK5	Q15078<br>Q00535	CHEMBL1907600	Kinase	0.538061807395	6 / 18
Cyclin-dependent kinase 1/cyclin B	CCNB3<br>CDK1<br>CCNB1<br>CCNB2	Q8WWL7<br>P06493<br>P14635<br>O95067	CHEMBL2094127	Other cytosolic protein	0.538061807395	4 / 9
Arginase-1 (by homology)	ARG1	P05089	CHEMBL1075097	Enzyme	0.538061807395	2 / 2
Cyclin-dependent kinase 6	CDK6	Q00534	CHEMBL2508	Kinase	0.498513190462	3 / 4
Cyclin-dependent kinase 2	CDK2	P24941	CHEMBL301	Kinase	0.498513190462	1 / 17
Tyrosinase	TYR	P14679	CHEMBL1973	Oxidoreductase	0.395895175206	2 / 3
Estradiol 17-beta-dehydrogenase 1	HSD17B1	P14061	CHEMBL3181	Enzyme	0.395895175206	8 / 4
Aryl hydrocarbon receptor	AHR	P35869	CHEMBL3201	Transcription factor	0.395895175206	1 / 1
Estrogen-related receptor alpha	ESRRA	P11474	CHEMBL3429	Nuclear receptor	0.395895175206	2 / 2
Beta amyloid A4 protein	APP	P05067	CHEMBL2487	Membrane receptor	0.261559713213	2 / 12
Poly [ADP-ribose] polymerase-1	PARP1	P09874	CHEMBL3105	Enzyme	0.261559713213	3 / 9
Transthyretin	TTR	P02766	CHEMBL3194	Secreted protein	0.261559713213	2 / 2
Matrix metalloproteinase 12	MMP12	P39900	CHEMBL4393	Protease	0.261559713213	1 / 1
Lymphocyte differentiation antigen CD38	CD38	P28907	CHEMBL4660	Enzyme	0.261559713213	2 / 3
Aldo-keto reductase family 1 member B10	AKR1B10	O60218	CHEMBL5983	Enzyme	0.261559713213	2 / 3
Tankyrase-2	TNKS2	Q9H2K2	CHEMBL6154	Enzyme	0.261559713213	4 / 12
Tankyrase-1	TNKS	O95271	CHEMBL6164	Enzyme	0.261559713213	4 / 28
DNA topoisomerase I (by homology)	TOP 1.00	P11387	CHEMBL1781	Isomerase	0.261559713213	1 / 1
Telomerase reverse transcriptase	TERT	O14746	CHEMBL2916	Enzyme	0.198389228663	9 / 22

Most likely targets for HA

Table 13. Most likely targets for HA

ATP-citrate synthase	ACLY	P53396	CHEMBL3720	Enzyme	0.765428310957	1 / 1
Squalene synthetase (by homology)	FDFT1	P37268	CHEMBL3338	Enzyme	0.0721804396226	0 / 1

Neuronal acetylcholine receptor protein alpha-7 subunit (by homology)	CHRNA7	P36544	CHEMBL2492	Ligand-gated ion channel	0.0517133123472	0 / 2
Egl nine homolog 1	EGLN1	Q9GZT9	CHEMBL5697	Oxidoreductase	0.0517133123472	0 / 2
HMG-CoA reductase	HMGCR	P04035	CHEMBL402	Oxidoreductase	0.0517133123472	0 / 1
Solute carrier family 22 member 6 (by homology)	SLC22A6	Q4U2R8	CHEMBL1641347	Electrochemical transporter	0.0312265582077	0 / 2
Protein farnesyltransferase	FNTA FNTB	P49354 P49356	CHEMBL2094108	Enzyme	0.0	0 / 5
Excitatory amino acid transporter 3	SLC1A1	P43005	CHEMBL2721	Electrochemical transporter	0.0	0 / 3
Prostanoid EP2 receptor	PTGER2	P43116	CHEMBL1881	Family A G protein-coupled receptor	0.0	0 / 1
Prostanoid FP receptor	PTGFR	P43088	CHEMBL1987	Family A G protein-coupled receptor	0.0	0 / 1
Niemann-Pick C1-like protein 1	NPC1L1	Q9UHC9	CHEMBL2027	Other membrane protein	0.0	0 / 2
Hydroxyacid oxidase 1	HAO1	Q9UJM8	CHEMBL4229	Enzyme	0.0	0 / 1
Lysine-specific demethylase 2A	KDM2A	Q9Y2K7	CHEMBL1938210	Eraser	0.0	0 / 1
Histone lysine demethylase PHF8	PHF8	Q9UPP1	CHEMBL1938212	Eraser	0.0	0 / 1
Lysine-specific demethylase 5C	KDM5C	P41229	CHEMBL2163176	Eraser	0.0	0 / 1
Metabotropic glutamate receptor 3	GRM3	Q14832	CHEMBL2888	Family C G protein-coupled receptor	0.0	0 / 5
Metabotropic glutamate receptor 2	GRM2	Q14416	CHEMBL5137	Family C G protein-coupled receptor	0.0	0 / 9
Glutamate receptor ionotropic kainate 1	GRIK1	P39086	CHEMBL1918	Ligand-gated ion channel	0.0	0 / 5
Glutamate receptor ionotropic, AMPA 1	GRIA1	P42261	CHEMBL2009	Ligand-gated ion channel	0.0	0 / 1
Adenosine A3 receptor	ADORA3	P0DMS8	CHEMBL256	Family A G protein-coupled receptor	0.0	0 / 1
Glutamate receptor ionotropic kainate 5	GRIK5	Q16478	CHEMBL2675	Ligand-gated ion channel	0.0	0 / 1
Metabotropic glutamate receptor 4	GRM4	Q14833	CHEMBL2736	Family C G protein-coupled receptor	0.0	0 / 3

Glutamate receptor ionotropic, AMPA 4	GRIA4	P48058	CHEMBL3190	Ligand-gated ion channel	0.0	0 / 1
Metabotropic glutamate receptor 5	GRM5	P41594	CHEMBL3227	Family C G protein-coupled receptor	0.0	0 / 2
Metabotropic glutamate receptor 8	GRM8	O00222	CHEMBL3228	Family C G protein-coupled receptor	0.0	0 / 3
Glutamate receptor ionotropic kainate 2	GRIK2	Q13002	CHEMBL3683	Ligand-gated ion channel	0.0	0 / 4
Glutamate receptor ionotropic kainate 3	GRIK3	Q13003	CHEMBL3684	Ligand-gated ion channel	0.0	0 / 1
Metabotropic glutamate receptor 1	GRM1	Q13255	CHEMBL3772	Family C G protein-coupled receptor	0.0	0 / 2
Metabotropic glutamate receptor 7	GRM7	Q14831	CHEMBL3777	Family C G protein-coupled receptor	0.0	0 / 1
Glutamate receptor ionotropic, AMPA 2	GRIA2	P42262	CHEMBL4016	Ligand-gated ion channel	0.0	0 / 1
Metabotropic glutamate receptor 6	GRM6	O15303	CHEMBL4573	Family C G protein-coupled receptor	0.0	0 / 3
UDP-glucuronosyltransferase 2B7	UGT2B7	P16662	CHEMBL4370	Enzyme	0.0	0 / 4
Glutamate carboxypeptidase II	FOLH1	Q04609	CHEMBL1892	Protease	0.0	0 / 7
6-phosphogluconate dehydrogenase	PGD	P52209	CHEMBL3404	Enzyme	0.0	0 / 2
Fatty acid binding protein adipocyte	FABP4	P15090	CHEMBL2083	Fatty acid binding protein family	0.0	0 / 1
Peroxisome proliferator-activated receptor alpha	PPARA	Q07869	CHEMBL239	Nuclear receptor	0.0	0 / 3
Fatty acid binding protein muscle	FABP3	P05413	CHEMBL3344	Fatty acid binding protein family	0.0	0 / 1
Fatty acid binding protein epidermal	FABP5	Q01469	CHEMBL3674	Fatty acid binding protein family	0.0	0 / 1
Peroxisome proliferator-activated receptor delta	PPARD	Q03181	CHEMBL3979	Nuclear receptor	0.0	0 / 2
Free fatty acid receptor 1	FFAR1	O14842	CHEMBL4422	Family A G protein-coupled receptor	0.0	0 / 1
Fatty acid binding protein intestinal	FABP2	P12104	CHEMBL4879	Fatty acid binding protein family	0.0	0 / 1
Bile acid receptor FXR	NR1H4	Q96RI1	CHEMBL2047	Nuclear receptor	0.0	0 / 7

Glucose-6-phosphate 1-dehydrogenase	G6PD	P11413	CHEMBL5347	Enzyme	0.0	0 / 5
G-protein coupled bile acid receptor 1	GPBAR1	Q8TDU6	CHEMBL5409	Family A G protein-coupled receptor	0.0	0 / 13
Carnitine O-palmitoyltransferase 1, liver isoform (by homology)	CPT1A	P50416	CHEMBL1293194	Enzyme	0.0	0 / 4
11-beta-hydroxysteroid dehydrogenase 1	HSD11B1	P28845	CHEMBL4235	Enzyme	0.0	0 / 4
11-beta-hydroxysteroid dehydrogenase 2	HSD11B2	P80365	CHEMBL3746	Enzyme	0.0	0 / 1
Androgen Receptor	AR	P10275	CHEMBL1871	Nuclear receptor	0.0	0 / 4
Vitamin D receptor	VDR	P11473	CHEMBL1977	Nuclear receptor	0.0	0 / 1
Cytochrome P450 19A1	CYP19A1	P11511	CHEMBL1978	Cytochrome P450	0.0	0 / 3
GABA A receptor alpha-2/beta-2/gamma-2	GABRA2 GABRB2 GABRG2	P47869 P47870 P18507	CHEMBL2111413	Ligand-gated ion channel	0.0	0 / 1
Dual specificity phosphatase Cdc25A	CDC25A	P30304	CHEMBL3775	Phosphatase	0.0	0 / 2
Aldo-keto reductase family 1 member B10	AKR1B10	O60218	CHEMBL5983	Enzyme	0.0	0 / 1
DNA polymerase beta (by homology)	POLB	P06746	CHEMBL2392	Enzyme	0.0	0 / 1
Histone deacetylase 3	HDAC3	O15379	CHEMBL1829	Eraser	0.0	0 / 1
Excitatory amino acid transporter 2	SLC1A2	P43004	CHEMBL4973	Electrochemical transporter	0.0	0 / 2
Carbonic anhydrase I	CA1	P00915	CHEMBL261	Lyase	0.0	0 / 1
Carbonic anhydrase IX	CA9	Q16790	CHEMBL3594	Lyase	0.0	0 / 1
Aminopeptidase B	RNPEP	Q9H4A4	CHEMBL2432	Protease	0.0	0 / 1
Prostaglandin E synthase	PTGES	O14684	CHEMBL5658	Enzyme	0.0	0 / 1
Corticosteroid binding globulin	SERPINA6	P08185	CHEMBL2421	Secreted protein	0.0	0 / 3
Testis-specific androgen-binding protein	SHBG	P04278	CHEMBL3305	Secreted protein	0.0	0 / 9
Estradiol 17-beta-dehydrogenase 3	HSD17B3	P37058	CHEMBL4234	Enzyme	0.0	0 / 10
GABA-B receptor (by homology)	GABBR1	Q9UBS5	CHEMBL2064	Family C G protein-coupled receptor	0.0	0 / 1

Supplementary Material S2

KEGG pathways for DS3

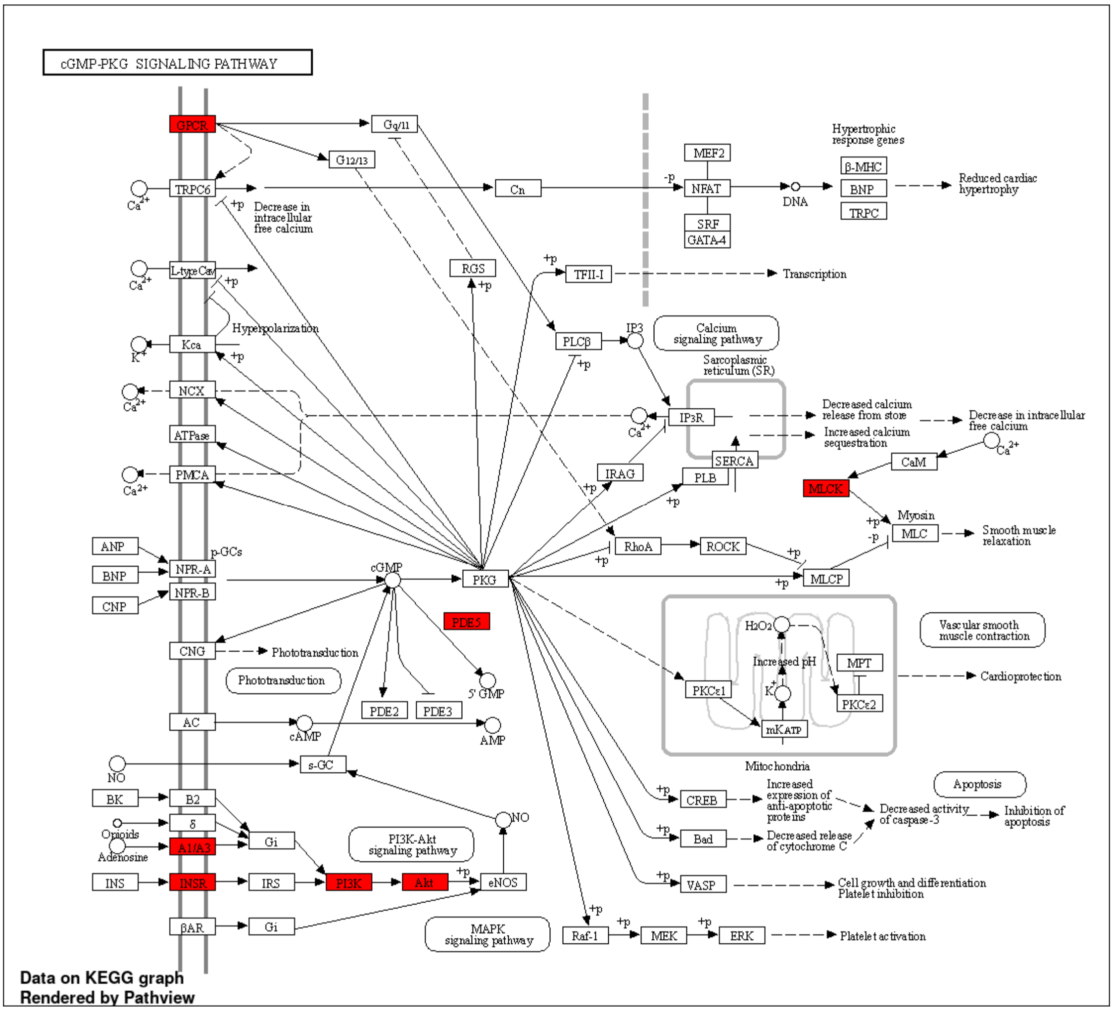

Figure 5. cGMP-Signalling patway with the targets of DS3 in red

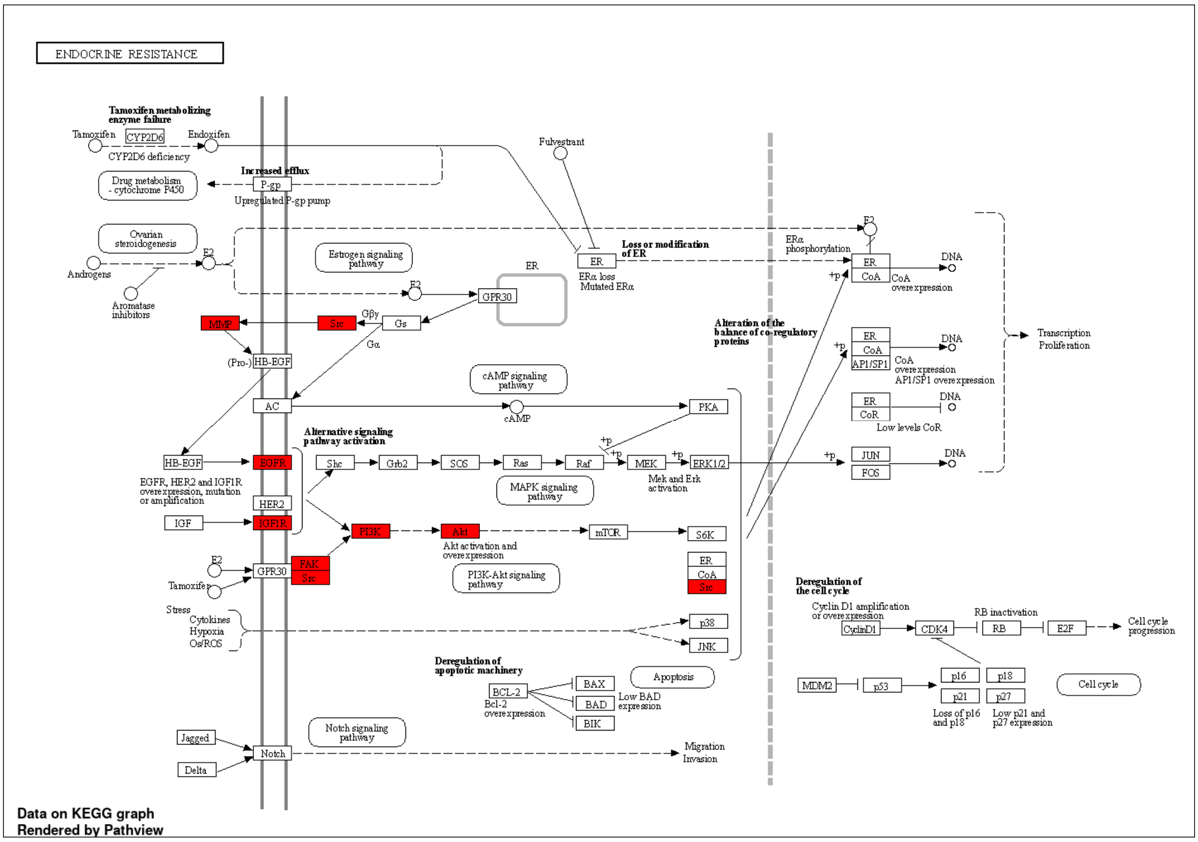

Figure 6. Endocrine Resistance pathway with the targets of DS3 in red

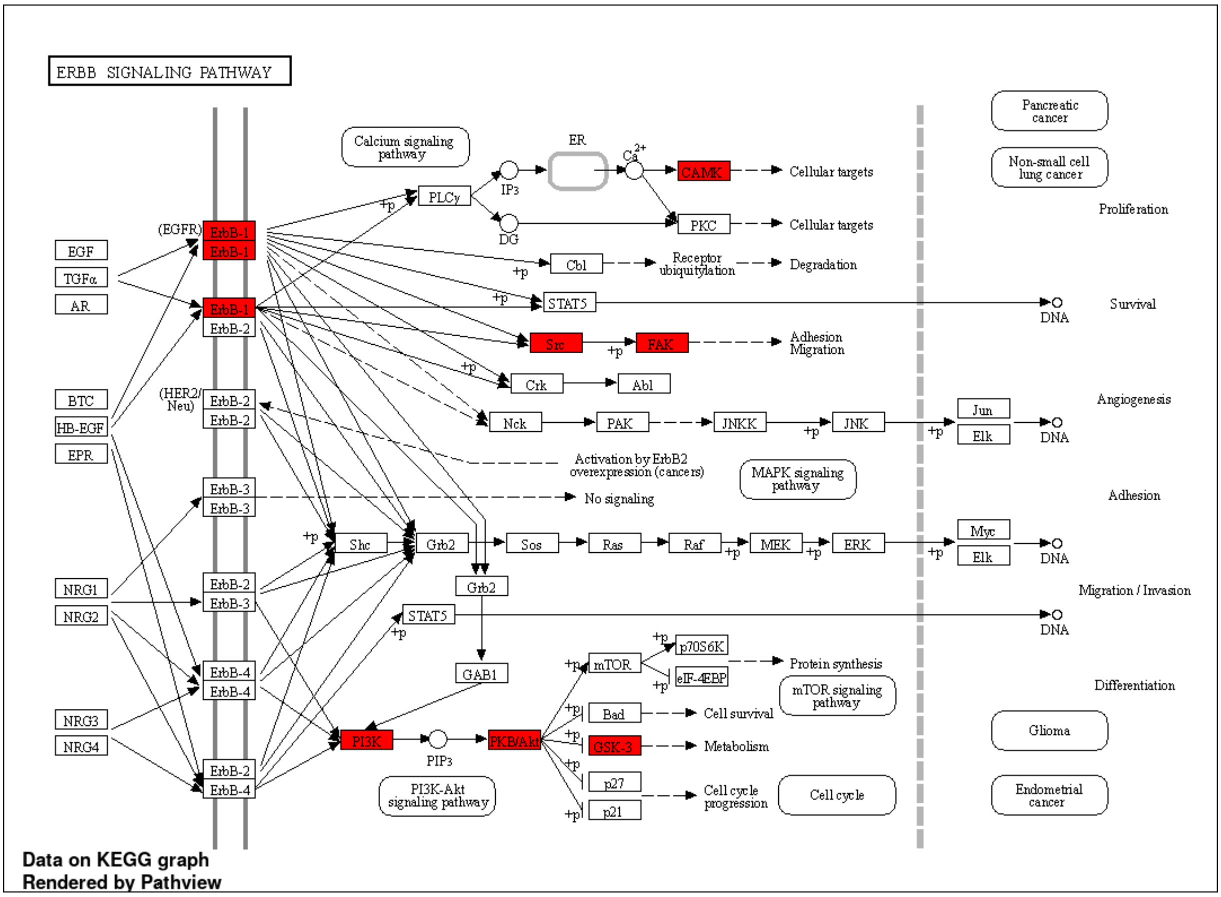

Figure 7. ERBB signaling pathway with the targets of DS3 in red

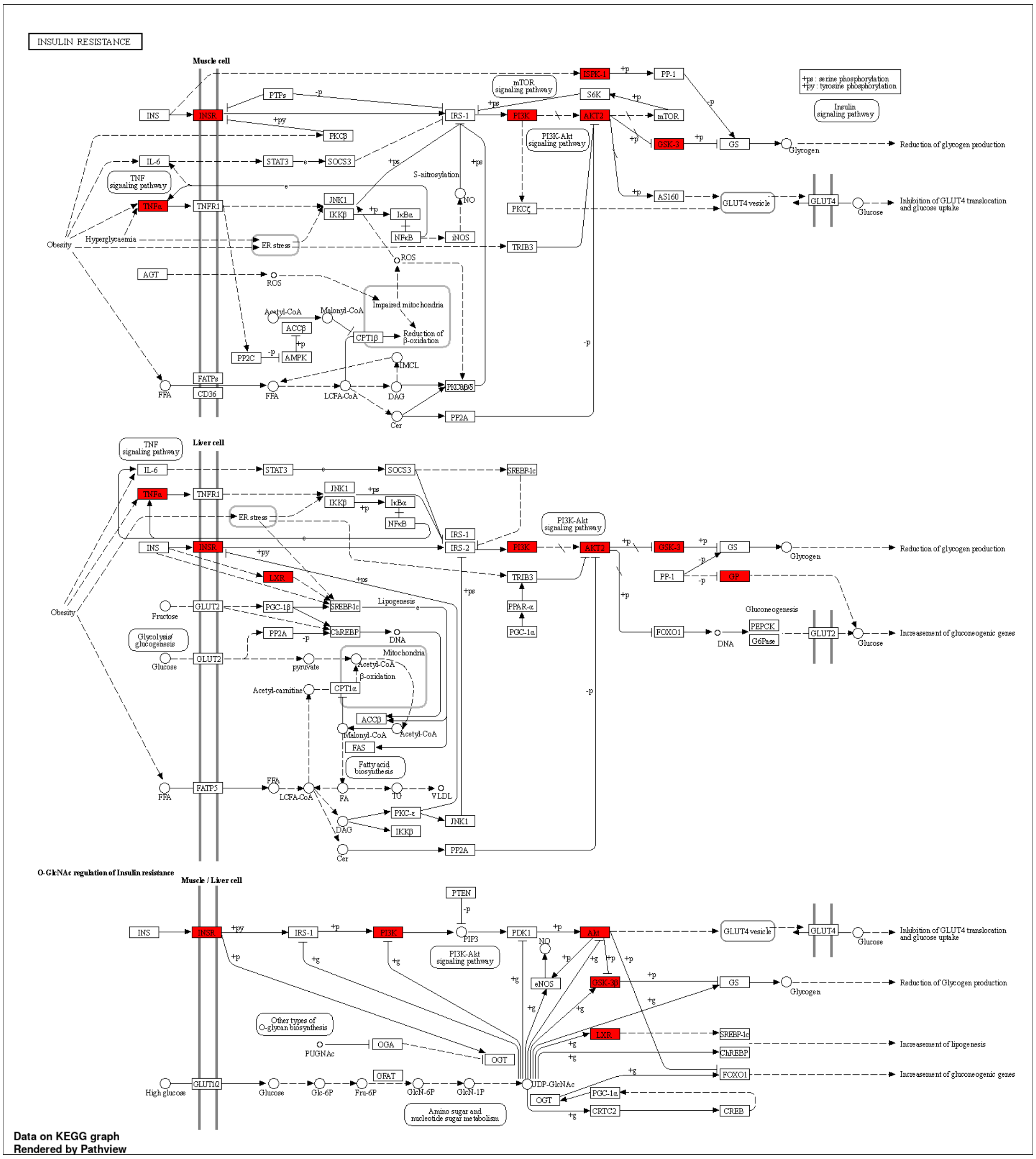

Figure 8. Insulin resistance pathways with the targets of DS3 in red

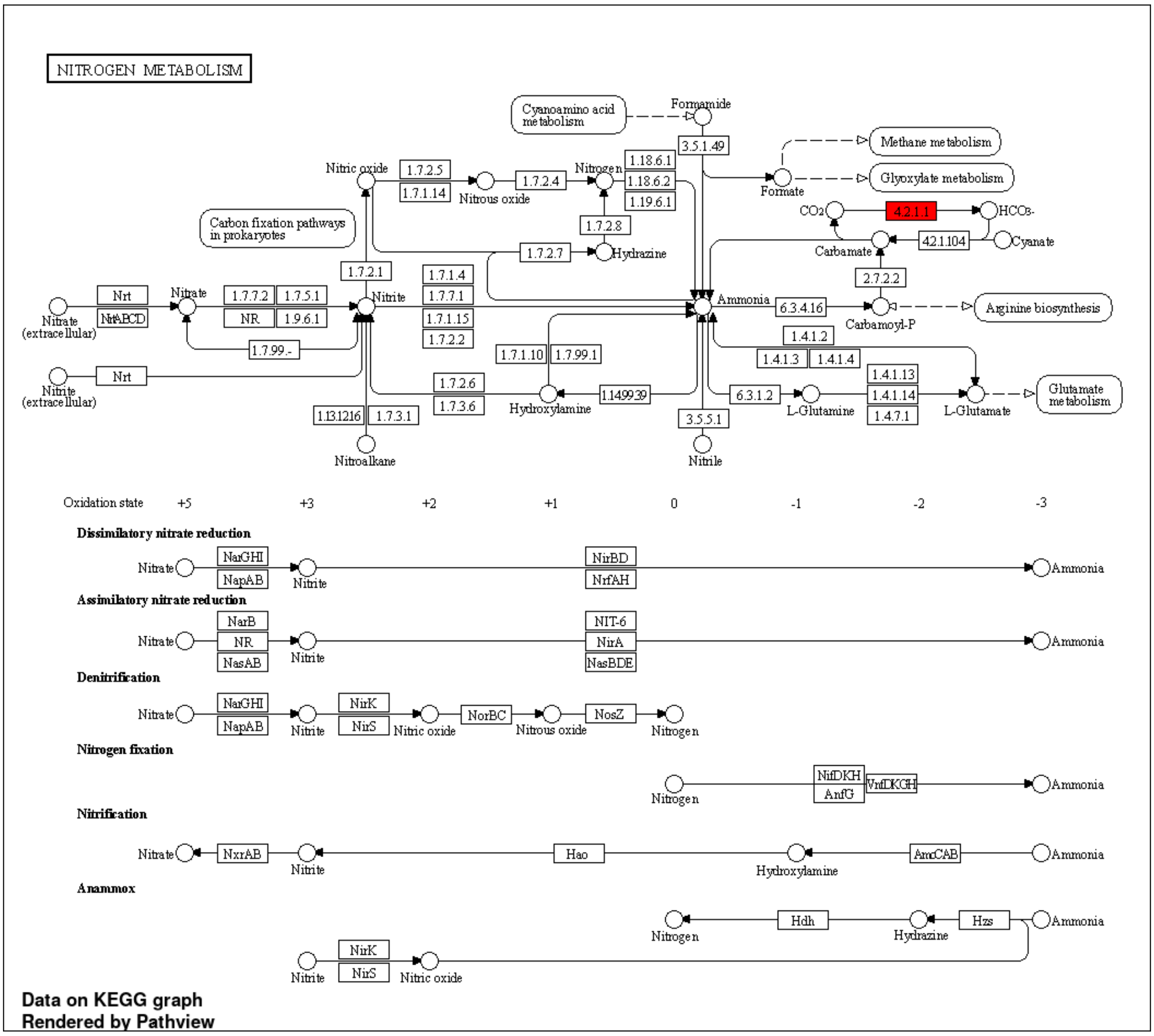

Figure 9. Nitrogen Metabolism pathway with the target of DS3 in red

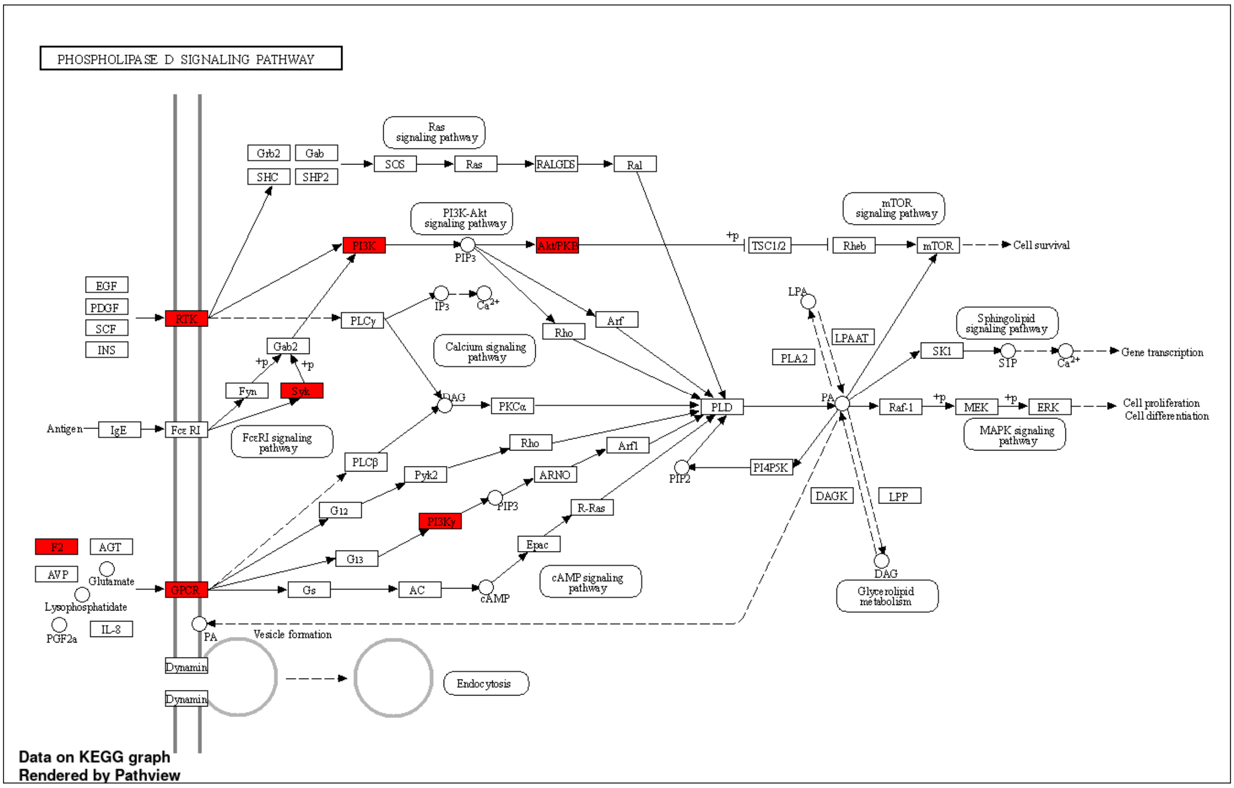

Figure 10. Phospholipase D signaling pathway with the targets of DS3 in red

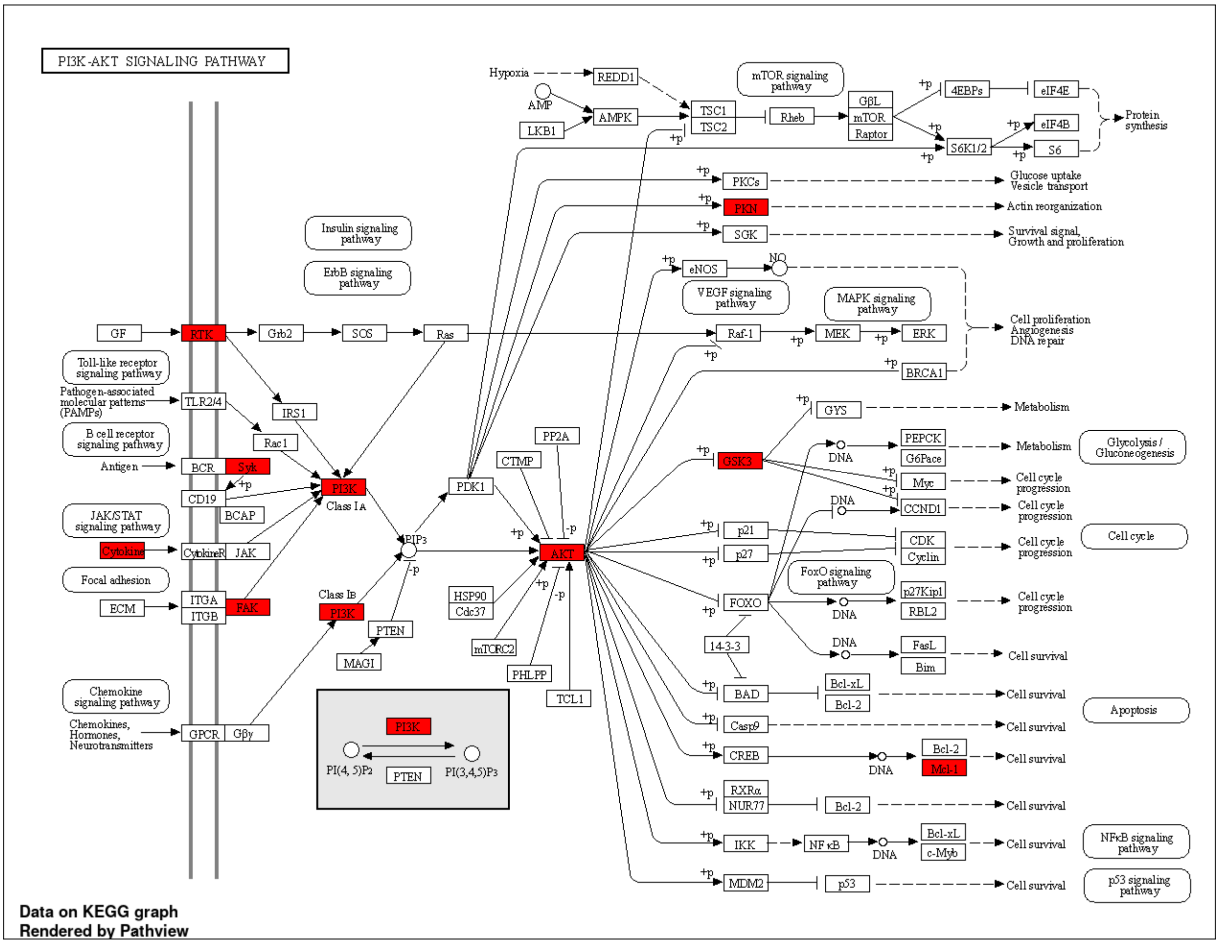

Figure 11. PI3K-AKT signaling pathway with the targets of DS3 in red

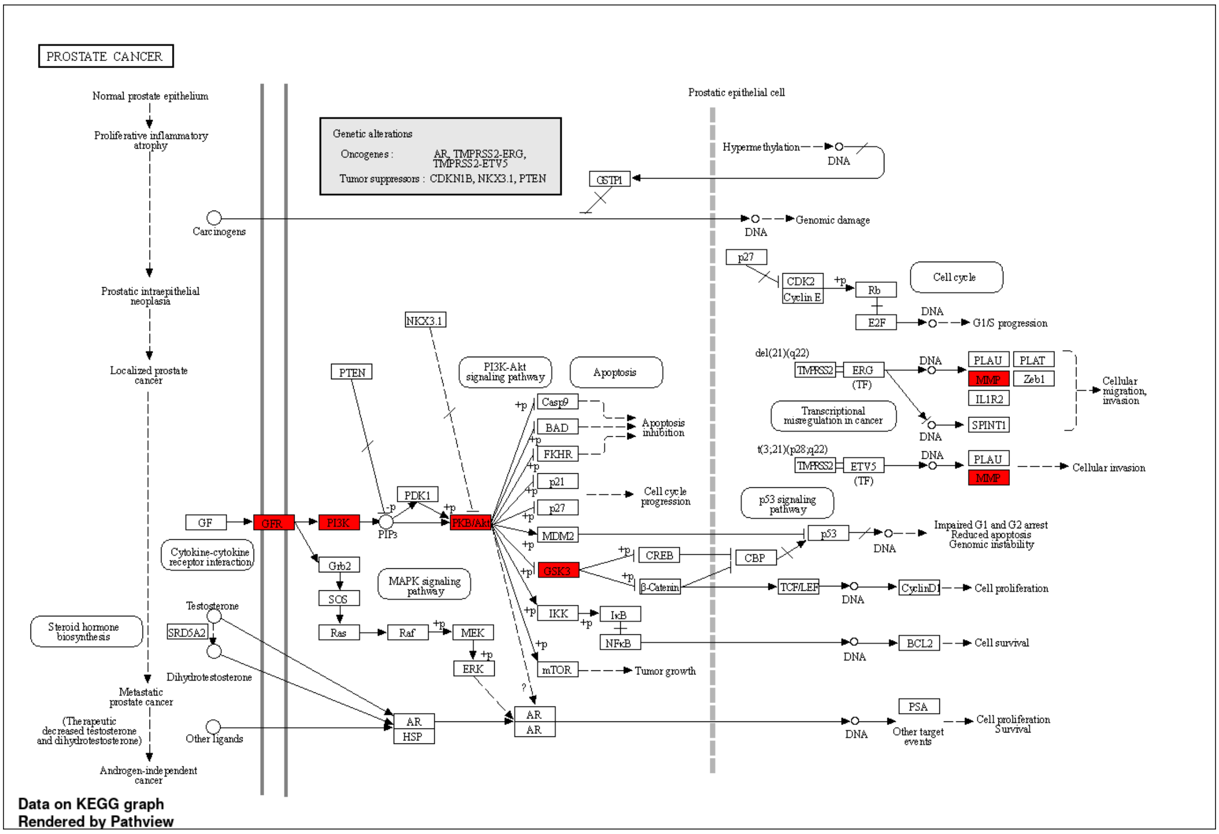

Figure 12. Prostate cancer pathways with the targets of DS3 in red

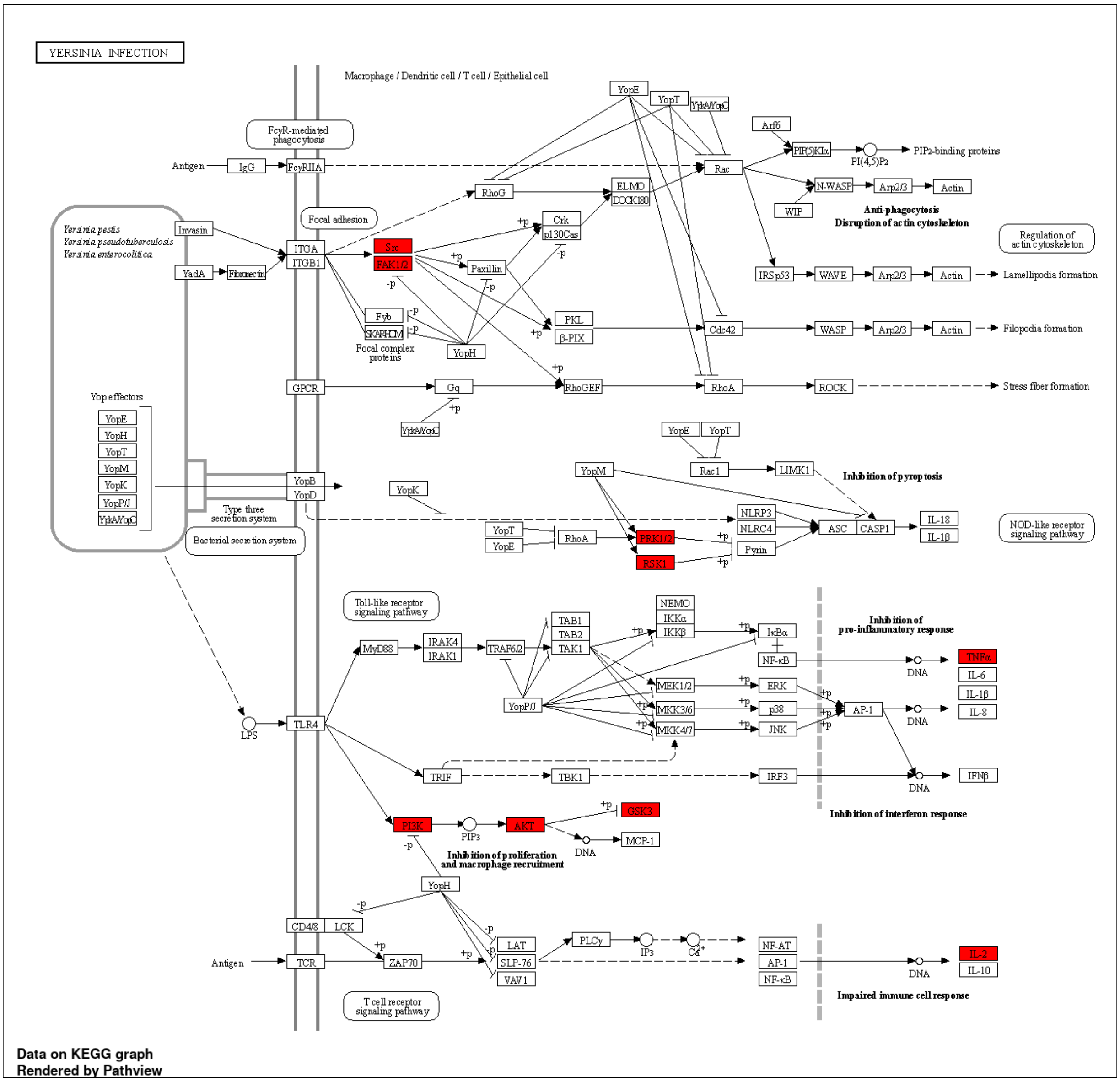

Figure 13. Yersenia Infection pathways with the targets of DS3 in red

KEGG pathways for QRC

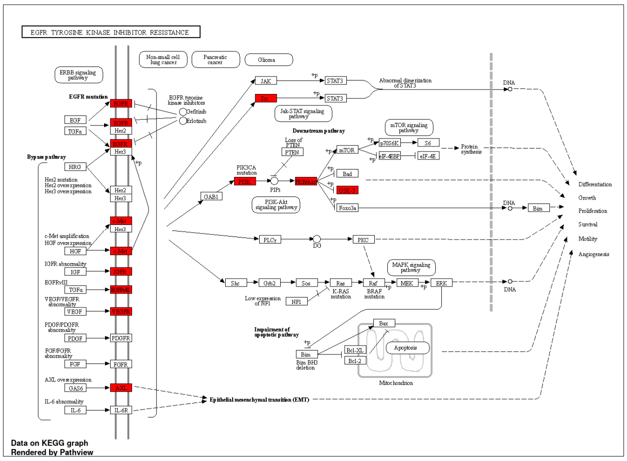

Figure 14. EGFR tyrosine kinase inhibitor resistance with the QRC targets in red

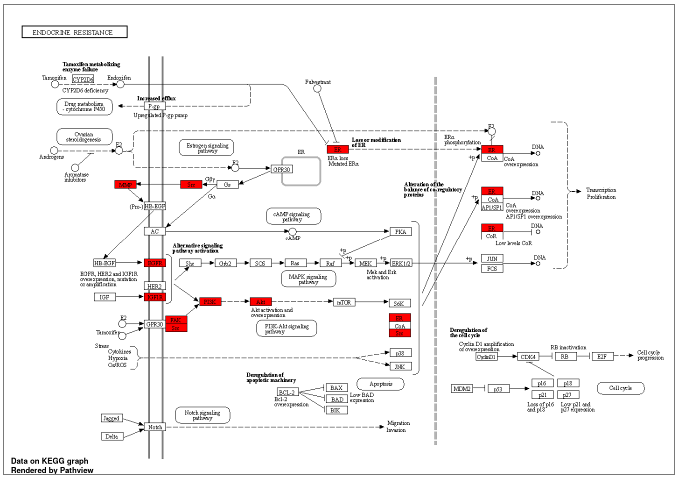

Figure 15. Endocrine resistance pathways with the QRC targets in red

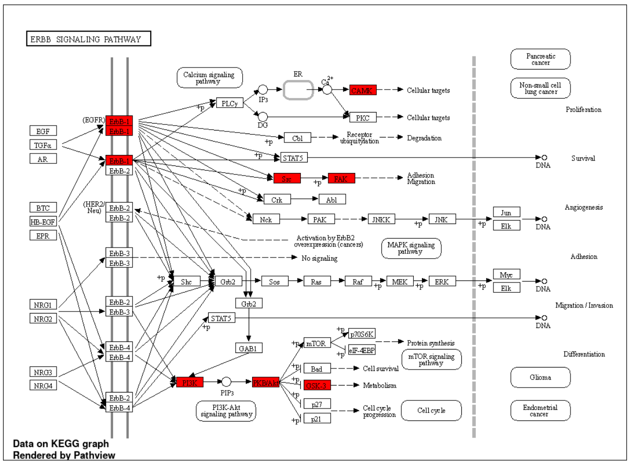

Figure 16. ERBB signaling pathway with the QRC targets in red

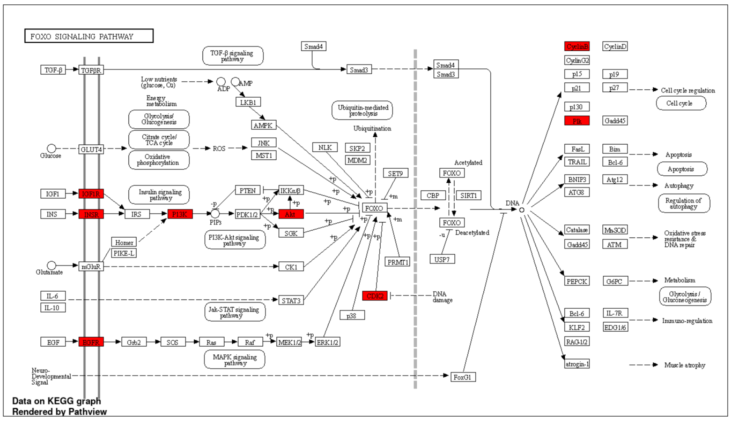

Figure 17. FOXO signaling pathway with the QRC targets in red.

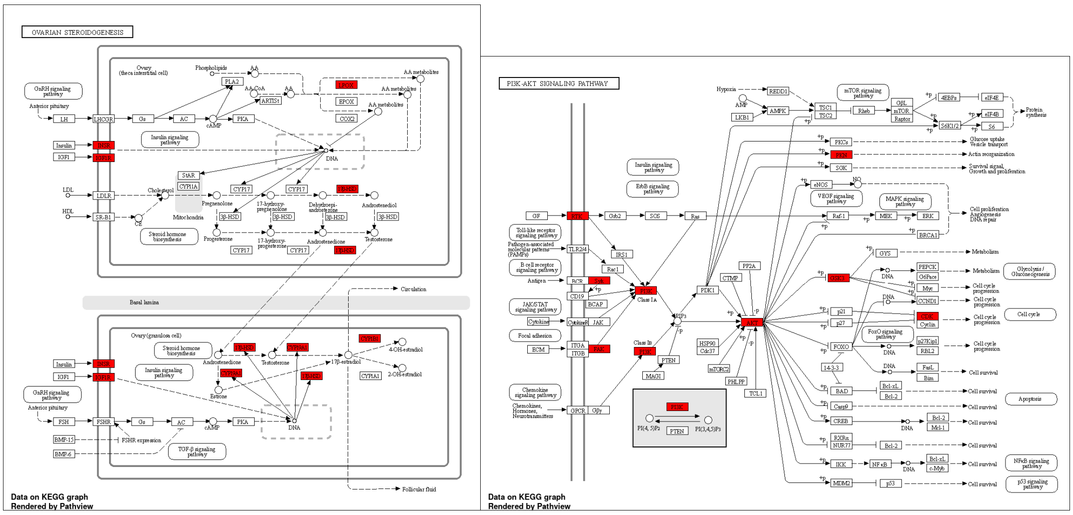

Figure 18. Ovarian steroidogenesis and PI3K-AKT signaling pathways with the QRC targets in red

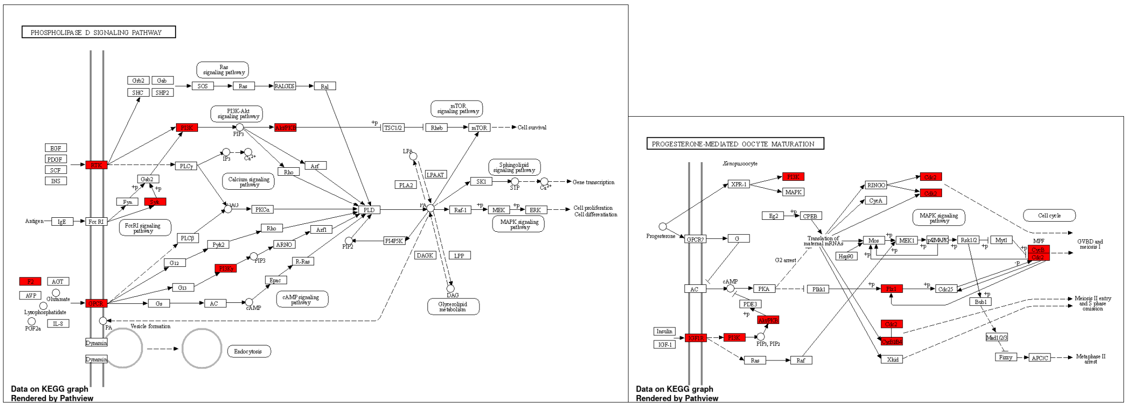

Figure 19. Phospholipase D signaling pathway and Progesterone mediated oocyte maduration with the QRC targets in red

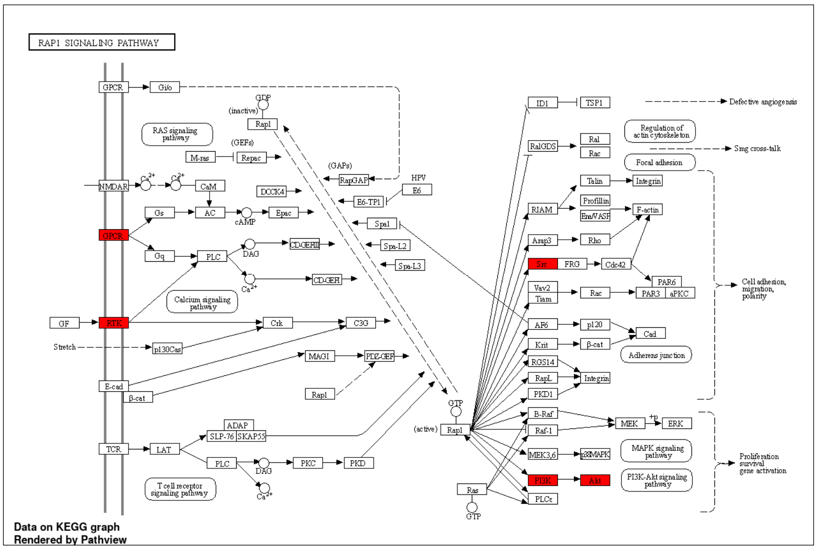

Figure 20. RAP1 signaling pathway with the QRC targets in red

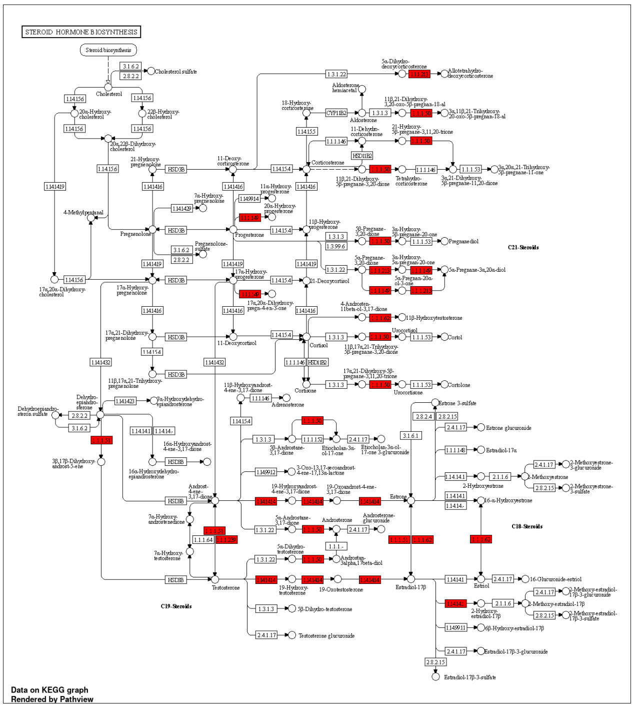

Figure 21. Steroid Hormone Biosynthesis with the QRC targets in red

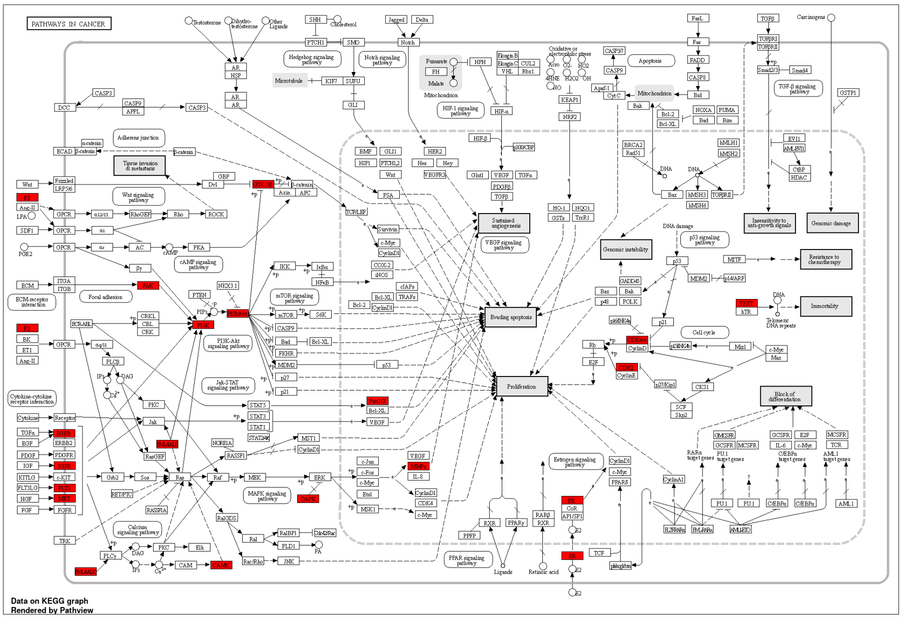

Figure 22. Pathways in cancer with the QRC targets in red

KEGG pathways for HA

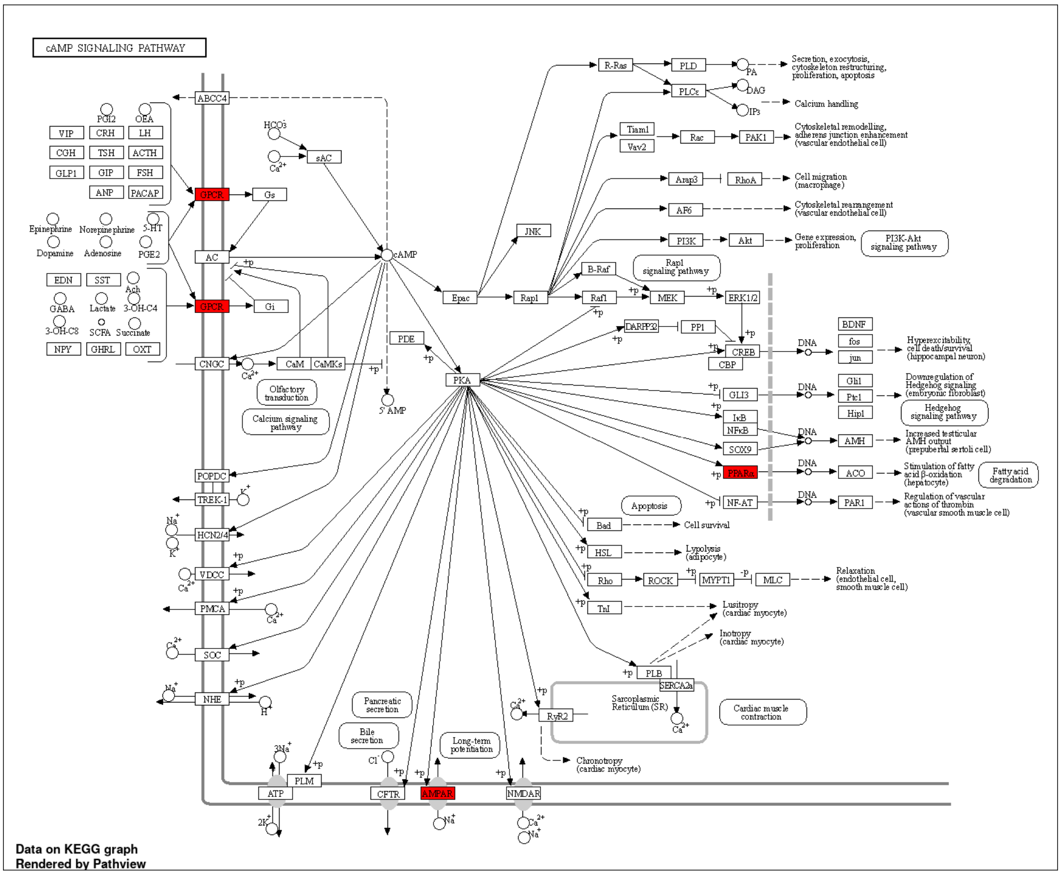

Figure 23. cAMP signaling pathway with the HA targets in red

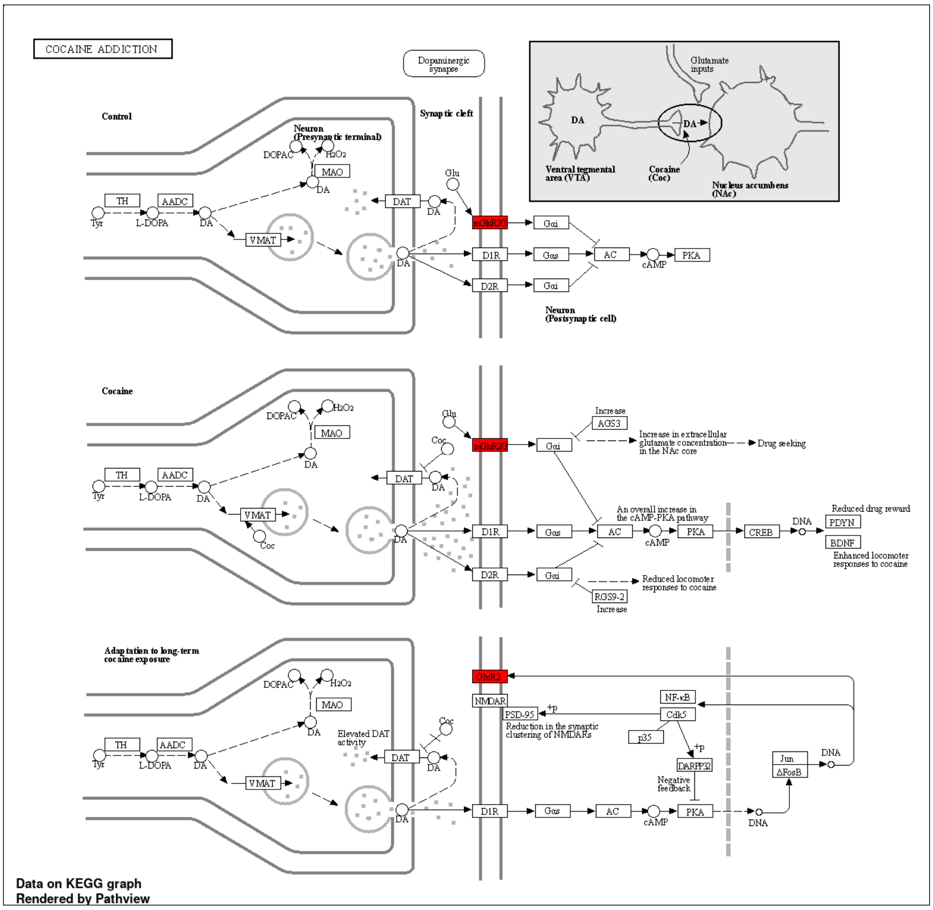

Figure 24. Cocaine addiction pathways with the HA targets in red

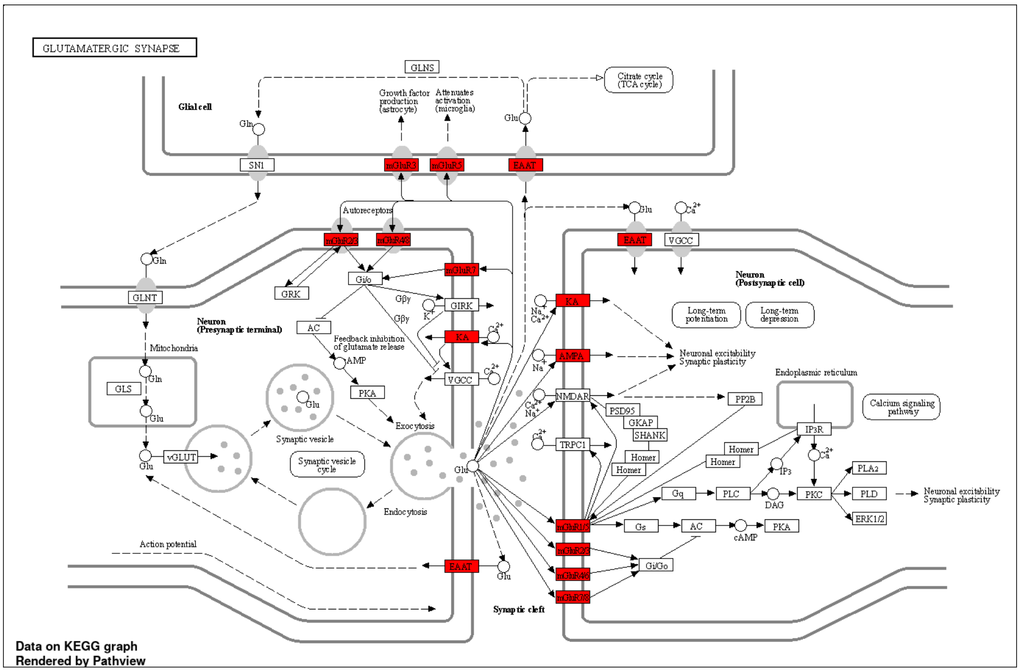

Figure 25. Glutamatergic synapse pathway with the HA targets in red

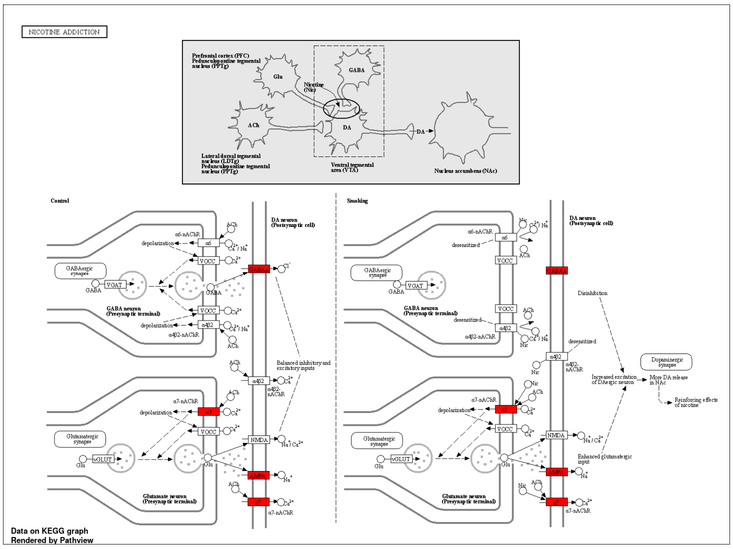

Figure 26. Nicotine addiction pathway with the HA targets in red

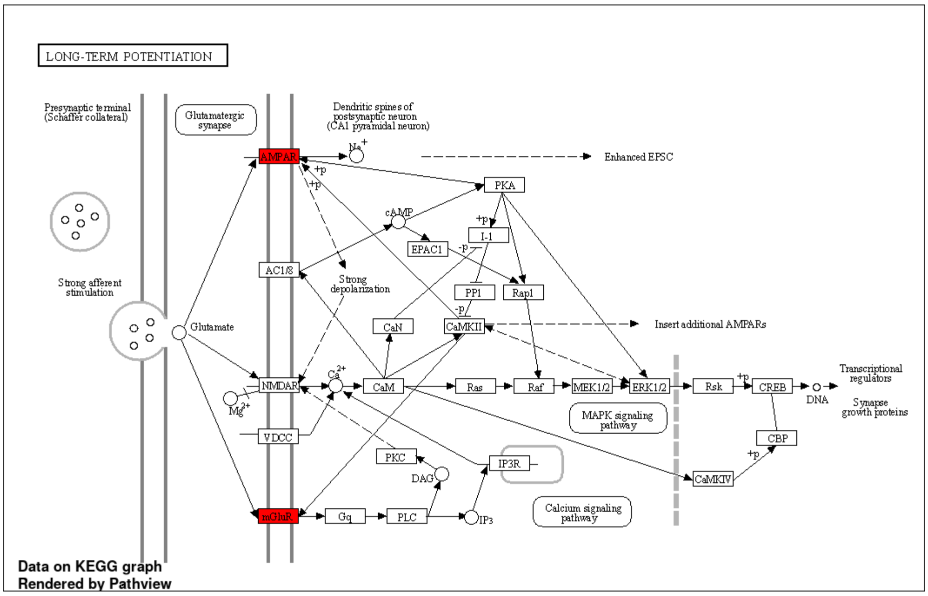

Figure 27. Long Term Potentiatoin with the HA targets in red

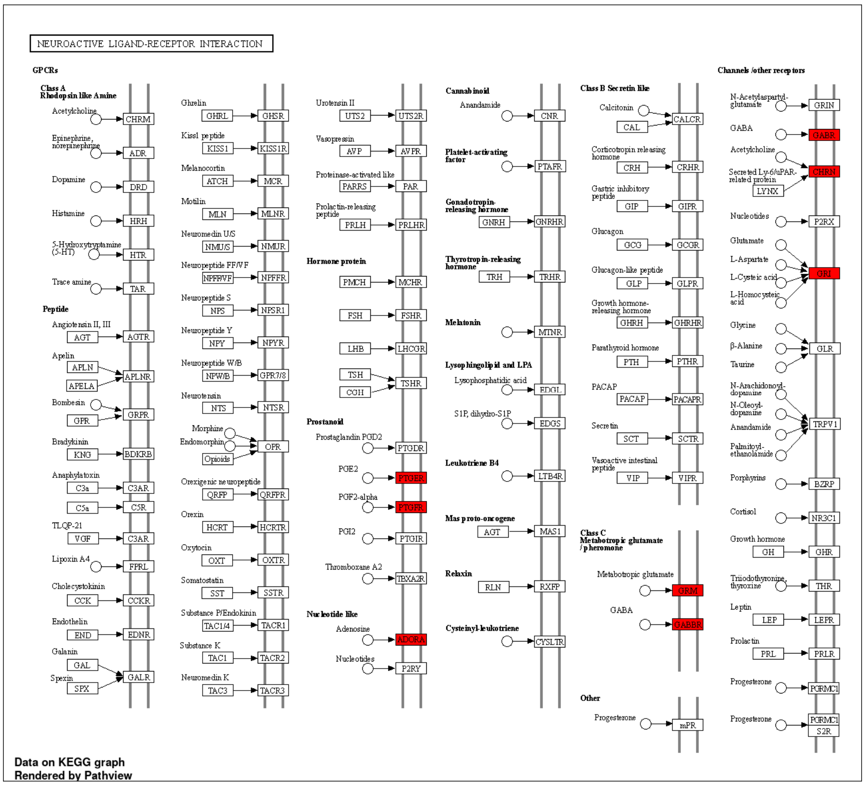

Figure 28. Neuroactive ligand-receptor interaction with the HA targets in red

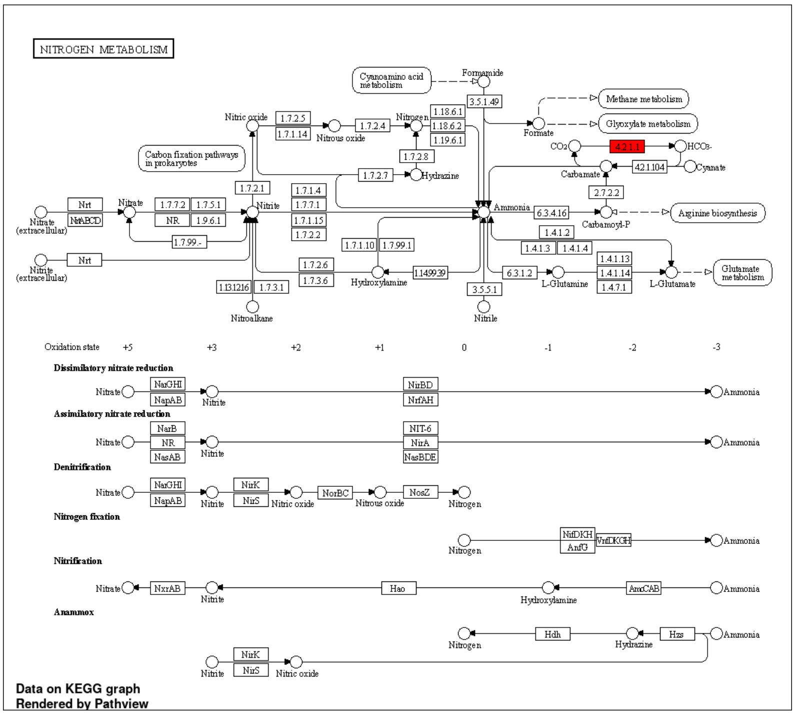

Figure 29. Nitrogen metabolism with the HA targets in red

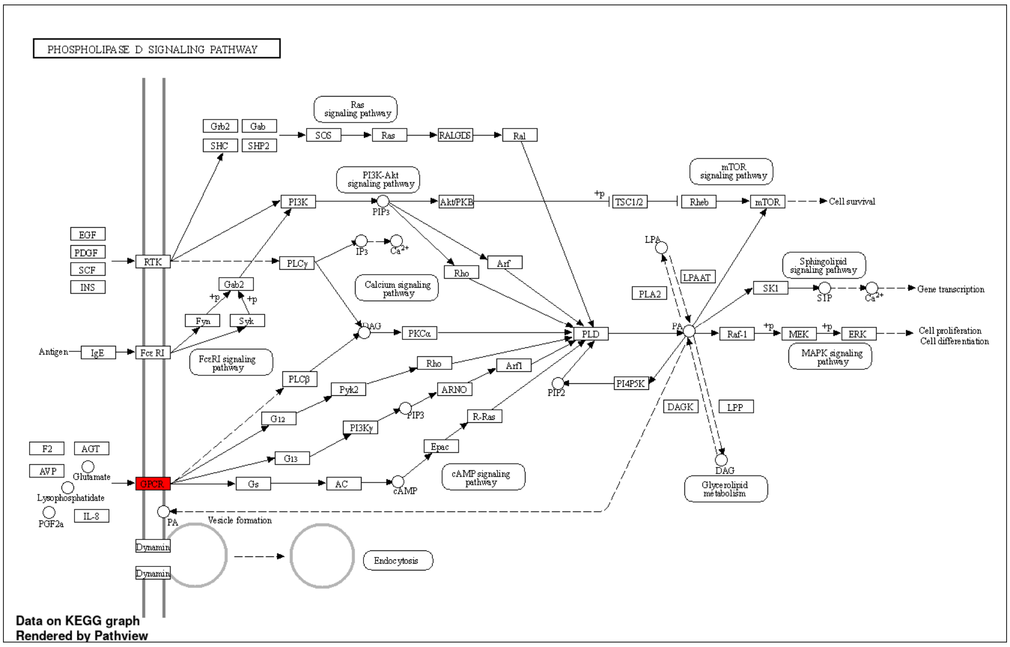

Figure 30. Phospholipase D Signaling pathway with the HA targets in red

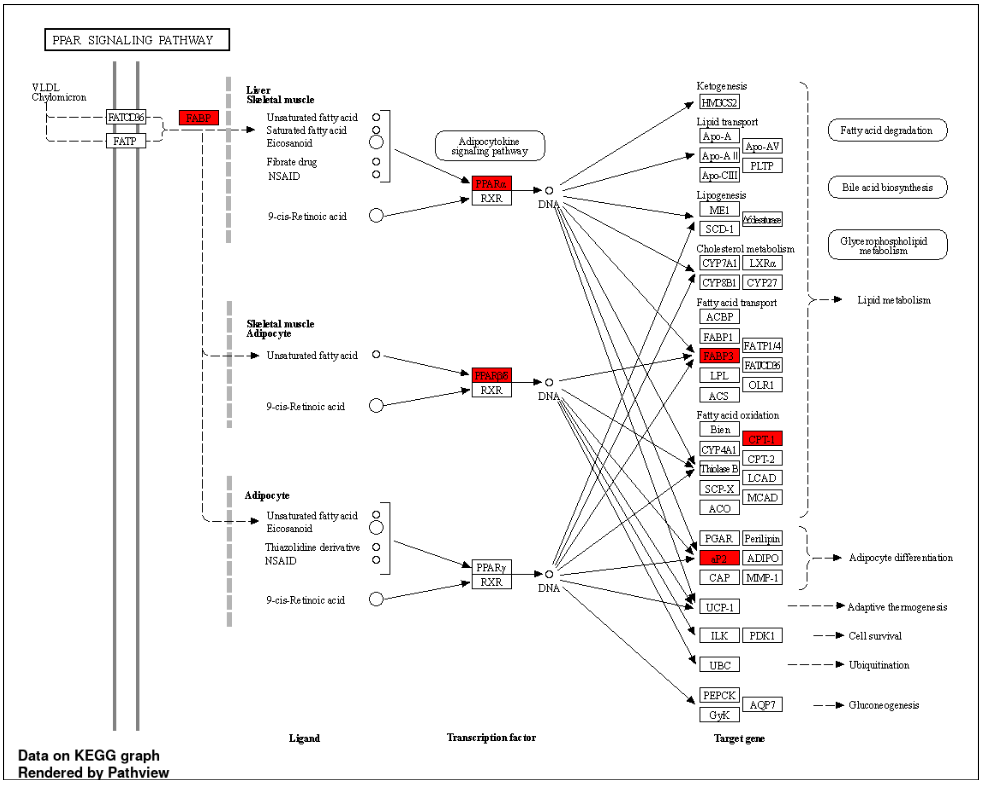

Figure 31. PPAR signaling pathway with the HA targets in red

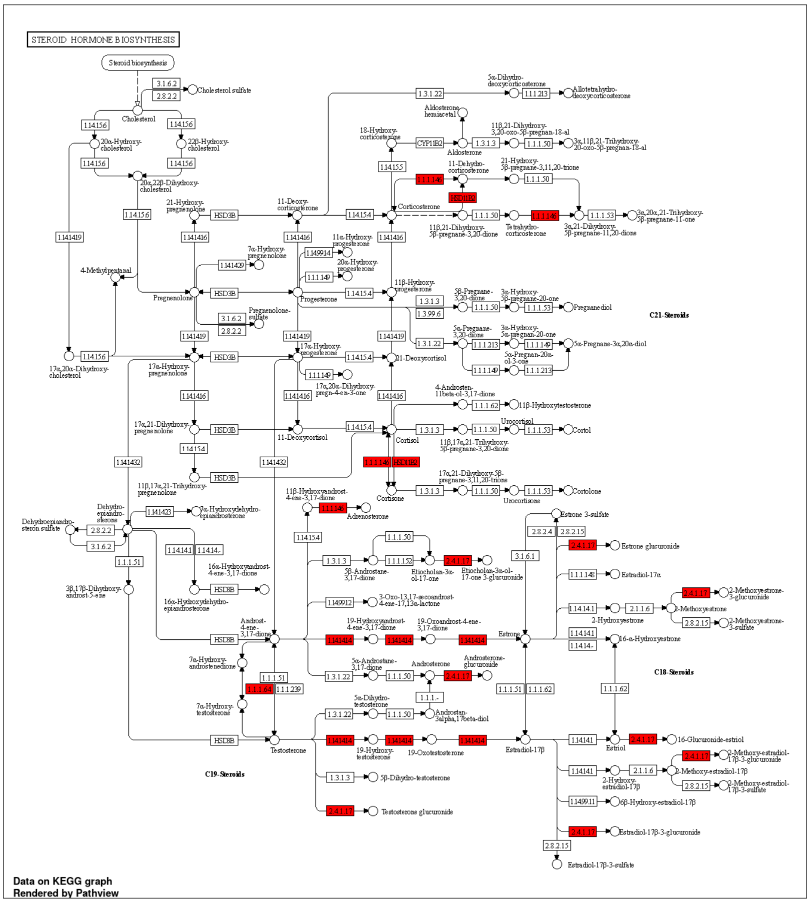

Figure 32. Steroid hormone biosynthesis with the HA targets in red

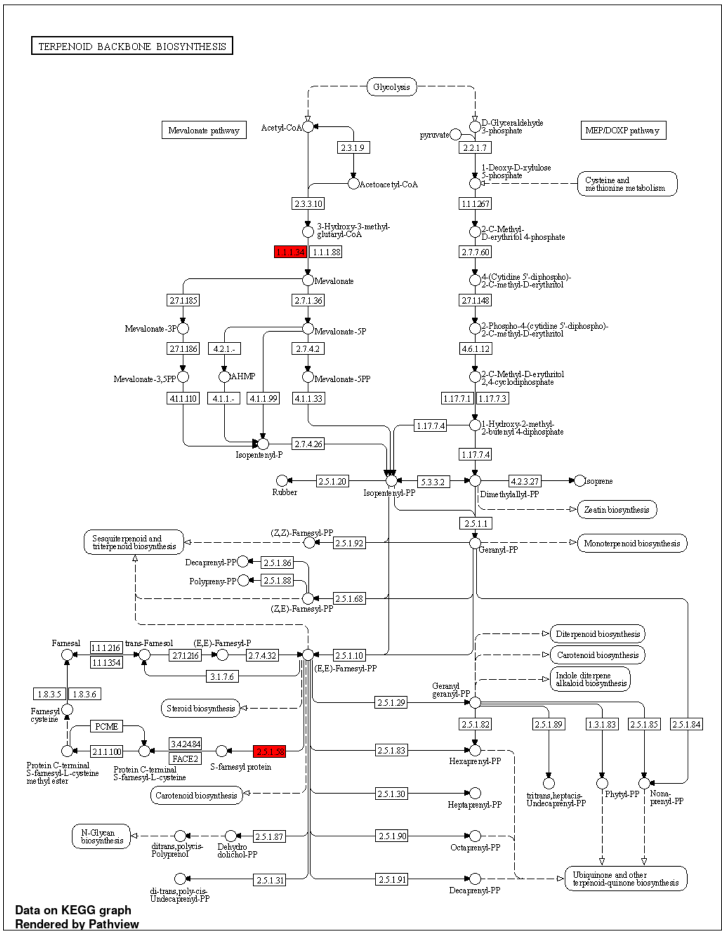

Figure 33. Terpenoid backbone biosynthesis with the HA targets in red
